# WA-YOLO: An explosive material detection algorithm for blasting sites based on YOLOv8

**DOI:** 10.1371/journal.pone.0318172

**Published:** 2025-04-22

**Authors:** LinNa Li, Han Gao, JunYi Lu, XiaoXiao Xu

**Affiliations:** 1 College of Science, Wuhan University of Science and Technology, Wuhan, Hubei, China; 2 Hubei Province Key Laboratory of Systems Science in Metallurgical Process, Wuhan, Hubei, China; 3 Hubei Province Intelligent Blasting Engineering Technology Research Center, Wuhan, Hubei, China; UO: University of Okara, PAKISTAN

## Abstract

Pyrotechnic detection has always been one of the critical issues in blasting safety. Due to the complex environment of blasting sites, irregular detonator wire postures, and the differences in object scales, making the detection of pyrotechnics more challenging. To address these challenges, this paper proposes an improved algorithm based on a multi-scale parallel attention mechanism and wavelet-separable convolution, called WA-YOLO. First, we integrate wavelet convolution into depthwise separable convolution and propose a novel convolutional block (WSDConv, Wavelet Separable Depthwise Convolution). This new convolutional block is added to the model’s backbone, improving feature extraction while also lowering computational parameters. Furthermore, we introduce an improved Cross Stage Partial (CSP) structure by combining multi-scale convolutions with a parallel attention mechanism, embedding it into the C2f module of the neck network to improve the model’s ability to detect objects of varying scales in complex backgrounds. To tackle the detection accuracy drop caused by the irregular shapes and varying aspect ratios of detonator wires, the model uses the Wise-IoU loss function. This enhances the model’s generalization and robustness by improving the precision of overlap calculations for bounding boxes. The experimental results show that the improved model achieved an average precision increase of 12.6% on the self-built dataset, particularly with an average precision increase of 8.3% in the detection of detonators. Additionally, the model performance also improved on the VOC2012 dataset, with a recall increase of 1.3% and an average precision increase of 1.6%. These results indicate that the proposed model exhibits strong generalization capabilities, can work effectively across different datasets, and provides an effective solution to the challenges of target detection in blasting environments.

## Introduction

Object detection in computer vision is essential in fields like security monitoring, medical diagnosis, and autonomous driving [1]. This technology has brought significant benefits to safety management at blasting sites. Several researchers have explored related applications based on intelligent video surveillance, such as using facial recognition for personnel identification and authentication at blasting sites [2], and developing safety management and warning systems based on the YOLOv3 deep learning algorithm [3,4]. These systems are capable of real-time monitoring and intelligent warning for the safety of personnel and equipment at open-pit mining blasting sites [5]. Although such research has made progress in intelligent video surveillance for blasting operations, challenges remain, such as limited accuracy in multi-scale object detection and vulnerability to background noise, indicating the need for further research on target detection in blasting environments.

We have observed that in the real-time detection of pyrotechnics at blasting sites, as the scale of the blasting surface expands, surveillance cameras capture broader views, leading to an increased detection distance. This increased distance reduces the size of objects in the captured images, complicating object detection [6]. This introduces certain challenges in detecting detonators in different postures in this paper. Furthermore, the task faces difficulties in multi-scale object detection. Baseline models tend to focus on local semantic information in shallow networks, and during feature extraction, smaller objects may be overshadowed by larger ones, potentially losing critical details that are essential for accurate detection [7].

To tackle these challenges, we present an efficient pyrotechnic detection algorithm for blasting sites. Our key contributions include:

1. Integration of Wavelet Convolution: We introduce wavelet convolution into depthwise separable convolution, proposing a new convolutional block (WSDConv). We replace certain standard convolutional blocks in the backbone network with WSDConv. Leveraging the properties of wavelet transforms, WSDConv effectively simulates large receptive fields, allowing it to separate important features in images. Compared to traditional depthwise separable convolution, WSDConv maintains high computational efficiency while offering superior performance in feature extraction and noise suppression.
2. Additionally, we propose an improved method that combines multi-scale feature extraction with attention mechanism fusion, integrating multi-scale convolution and parallel attention mechanisms into the C2f module of the neck network to create a new C2f-MM module. This method enhances the model’s detection capability for targets of varying scales and complex backgrounds through parallel channel attention and spatial attention, effectively improving the accuracy of small target detection and the robustness in complex scenes.
3. WIoU Loss Function: To address the decline in detection accuracy caused by the irregular shapes and large aspect ratio variations of detonator wires, we introduce the WIoU loss function. We comprehensively validate the WA-YOLO model on our custom-built pyrotechnic dataset and several public datasets. Results show that the proposed model achieves significant improvements in detection efficiency and accuracy. Furthermore, these results demonstrate the feasibility of introducing wavelet-based separable convolution and multi-scale feature extraction with attention mechanisms. The WA-YOLO algorithm offers practical solutions for pyrotechnic detection tasks at blasting sites.

## Related work

This section reviews and analyzes the current mainstream algorithm models and the key characteristics of the blasting site, providing theoretical support for the improved model proposed in this paper.

Mainstream object detection algorithms mainly use deep learning, classified into single-stage and two-stage models. In this study, we employ the YOLO series [8,9], a representative of single-stage detection algorithms. Compared to two-stage models, which process candidate frames through classifiers, single-stage methods offer higher detection speed, making them widely used in real-time object detection tasks. However, this speed often comes at the expense of some accuracy, prompting numerous researchers to design deeper networks to enhance the performance of single-stage models in various visual tasks [10].

Recent studies show that attention mechanisms are vital for capturing key information. Many researchers have attempted to integrate these mechanisms into deep CNNs to improve image classification performance [11]. For instance, SENet, proposed by Hu et al., enhances feature representation by exploring inter-channel relationships [12]. Wang et al. developed a residual attention network [13] that utilizes stacked encoder-decoder attention modules, showing not only superior performance but also robustness against noisy inputs. Despite the significant improvements in model accuracy achieved through attention mechanisms, they often introduce computational redundancy and increase model complexity. Howard et al. [14] introduced the MobileNets architecture, highlighting the advantages of depthwise separable convolutions in reducing model size and computational complexity. Shahaf E. Findere et al. [15] proposed an innovative convolution method based on wavelet transforms, providing convolutional neural networks (CNNs) with an effective means of simulating a global receptive field. This method significantly enhances model performance while reducing parameters and computational load. Although these studies have made notable progress in improving model accuracy and reducing computational overhead, challenges remain in handling the diversity of object scales in detection tasks.

In the practical application of blast detection, several challenges arise, including complex backgrounds, dynamic scene noise, target diversity, and scale variability. Wang et al. [16] adapted YOLOv8 for object detection in remote sensing images, targeting complex backgrounds and small, varied objects. They added a small object detection layer and used an EMA attention module to incorporate the C2f-E structure. Wang et al. [6] enhanced UAV-based YOLOv8 by using the Boromir attention mechanism to highlight important information and integrating the FFNB mechanism for effective multi-scale feature fusion. These strategies effectively enhance the detection of small objects in complex environments and provide valuable insights for research on multi-scale object detection.

Moreover, compared to applications in public safety and other fields, research on enhancing the detection of explosive materials at blasting sites through the integration of various deep learning algorithms and techniques remains relatively limited. Therefore, addressing the practical challenges of explosive material detection at blasting sites, we redesigned the depthwise separable convolution (WSDConv) using wavelet transforms and introduced multi-scale convolution and parallel attention mechanisms in the model’s feature fusion network to ensure that the model can tackle the challenges of detecting multi-scale objects at blasting sites.

## Introduction to the YOLOv8 network model

**Fig 1:**
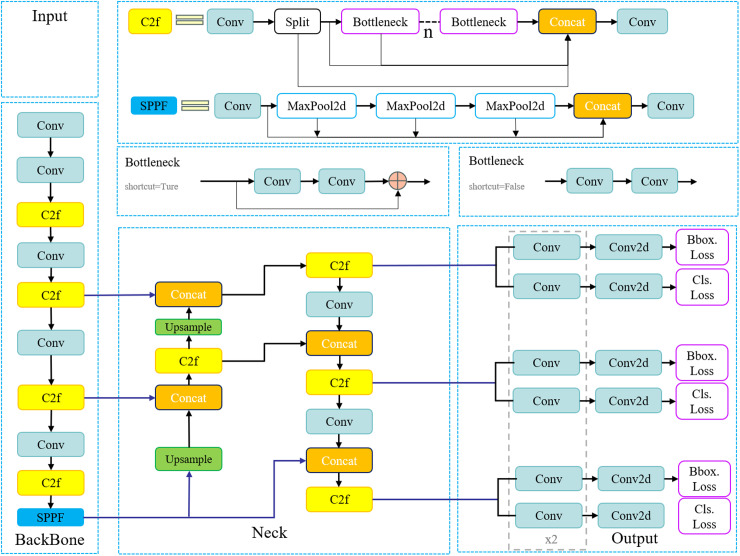
Structure of the YOLOv8 model.

As illustrated in Fig 1, the YOLOv8 model comprises three main components: the backbone network, the neck network, and the detection network.

### Backbone network

The YOLOv8 model utilizes the CSPDarknet53 [17] backbone network, which features five downsampling stages to extract multi-scale features. By utilizing the C2f module to replace the cross-stage partial (CSP) module previously used in the YOLO series [18], the C2f module is further divided into dense and residual structures. This design aims to balance the richness of feature extraction with computational efficiency while enhancing the network’s flexibility and training stability. Additionally, the backbone network includes an optimized Spatial Pyramid Pooling module (SPPF), which extracts contextual information at different scales through multi-scale pooling and feature concatenation, thereby optimizing feature representation [19].

### Neck network

During multi-scale feature fusion, the YOLOv8 model employs a top-down Feature Pyramid Network (FPN) [20] and a bottom-up Path Aggregation Network (PANet) [21]. By combining the PANet-FPN structure with the C2f module, the neck network significantly improves feature representation, producing feature maps at different scales while integrating shallow and deep information.

### Detection head network

YOLOv8 significantly improves the model’s accuracy and robustness by introducing a task-aligned allocator that replaces the traditional anchor box mechanism [22]. This allocator dynamically classifies samples as positive or negative, thereby enhancing the model’s precision in object detection tasks. Additionally, the detection head network uses a decoupled structure, separating target classification from bounding box regression. It employs binary cross-entropy loss (BCE loss) for classification and combines Distribution Focal Loss (DFL) [23] with Complete Intersection over Union (CIoU) [24] loss for regression. These efficient loss functions enable YOLOv8 to optimize precise object localization, boosting overall performance.

## Model and methods

To address the detection challenges posed by the complex environment of blasting sites, the diversity of target scales, and the varying orientations of detonator leads, we propose several key enhancements to the model.

First, we improve upon the traditional depthwise separable convolution by introducing a novel convolution block called WSDConv, which is integrated into the backbone network. This enhancement increases the network’s feature extraction capabilities and effectively reduces the risks of both missed detections and false positives, allowing for more accurate detection of explosive materials at blasting sites. Second, we propose the C2f-MM module, which builds upon the original C2f module by integrating multi-scale convolutions, as well as parallel channel attention and spatial attention modules. This design enables the model to dynamically emphasize key channel and spatial locations during feature extraction, effectively capturing information at various scales. As a result, the model’s detection accuracy for explosive materials at blasting sites in complex scenes is significantly improved. Finally, the Weighted Intersection over Union (WIoU) metric is introduced to enhance the model’s ability to correct for offset boxes by incorporating a weighting factor. This improvement ensures more precise localization of detected objects, effectively reducing errors in the detection of explosive materials.

**Fig 2:**
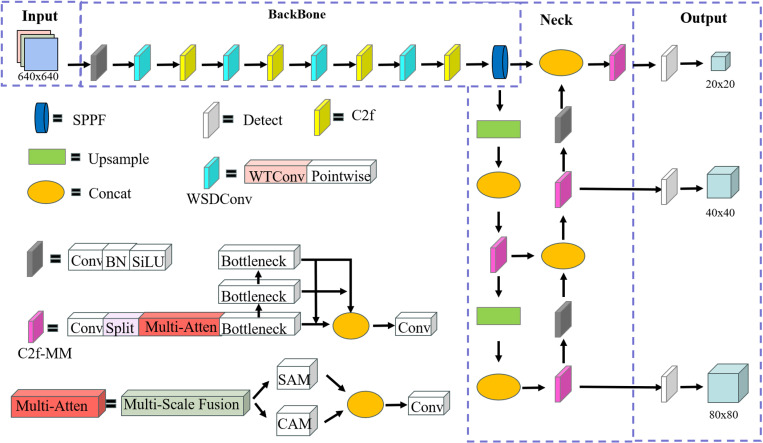
Structure of WA-YOLO.

These enhancements greatly boost the model’s detection efficiency and robustness. The specific improvements to the network structure are shown in Fig 2.

The workflow of the WA-YOLO model is primarily divided into three parts: input processing, feature extraction and object detection, and post-processing and output. In real-time explosive item detection at blasting sites, the process begins with preprocessing the raw images. This includes resizing the images (e.g., to 640x640 pixels) to meet the model’s input requirements, followed by image normalization to scale the pixel values within the range [0,1]. Data augmentation techniques such as flipping, rotating, and cropping are then applied to generate diversified training samples.

Next, feature extraction is performed using the WSDConv module, which integrates the properties of wavelet transforms to enhance the feature representation capability. During feature extraction, the C2f-MM module further improves the model’s ability to adapt to complex scenes and multi-scale targets through multi-scale convolutions and parallel channel and spatial attention mechanisms.

Finally, the WIoU loss function is employed to precisely correct the offset boxes, significantly improving the localization accuracy of irregular targets, such as detonators. The model output undergoes non-maximum suppression (NMS) to remove redundant boxes and further classifies and locates the targets, ensuring the accuracy of the detection results. These innovations provide strong support for the precise detection of explosive items at blasting sites. Through these design advancements, WA-YOLO achieves efficient explosive item detection in complex backgrounds while maintaining strong robustness.

### Wavelet separable depthwise convolution

**Fig 3:**
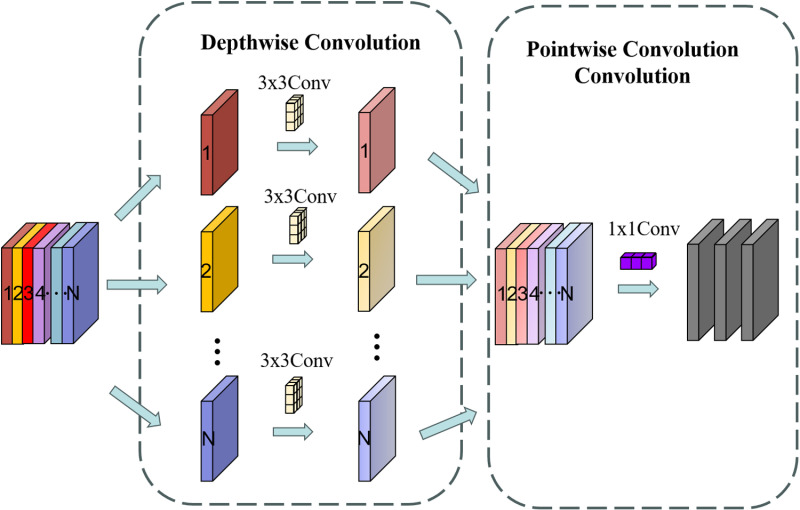
Structure of depthwise separable convolution.

In the backbone network of YOLOv8, the basic convolution block is the core unit for feature extraction, primarily consisting of standard convolution layers, batch normalization, and activation functions. Standard convolution performs a pixel-wise weighted sum operation on the input feature map using convolution kernels to extract features at different scales and spatial locations. However, despite its excellent performance in feature extraction, standard convolution suffers from issues such as a large number of computational parameters and a limited receptive field. To address this, we attempt to replace some of the basic convolution blocks in the backbone network with depthwise separable convolutions. This method decomposes the standard convolution into two steps: depthwise convolution and pointwise convolution, significantly reducing the number of computational parameters. The structure of depthwise separable convolution is illustrated in Fig 3.

**Fig 4:**
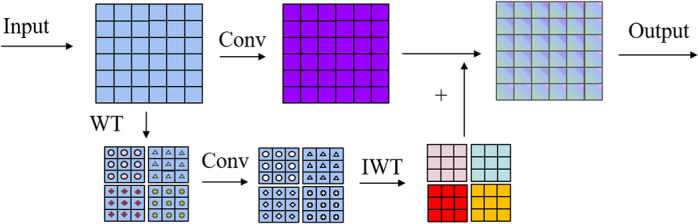
Operational principle of wavelet transform convolution.

During the implementation of depthwise separable convolutions, we found that its receptive field for feature extraction is limited, making it difficult to effectively capture global features. Additionally, it performs inadequately in extracting details and edge information, which are essential for detecting explosive materials in complex scenes. Therefore, we introduce wavelet convolution, which enhances the model’s receptive field and its ability to capture global information through multi-frequency decomposition via wavelet transforms. The operational principle of wavelet transform convolution (WTConv) using a first-level wavelet decomposition is illustrated in Fig 4.

Here, WT represents the wavelet transform, and IWT represents the inverse wavelet transform. Wavelet transform is a method for decomposing signals into different frequency bands, allowing the input signal to be separated into low-frequency and high-frequency components. Wavelet transform convolution (WTConv) utilizes this characteristic to separate the frequency band features of the input image and perform convolution operations on each frequency band separately. Subsequently, the processed high-frequency and low-frequency components are reconstructed back to the original space through inverse wavelet transformation, generating the final output features. The Haar single-level one-dimensional wavelet transform can be represented by the following formula:


$$X_{LL},X_{LH},X_{HL},X_{HH}=Conv(\left[f_{LL},f_{LH},f_{HL},f_{HH}\right],X)$$ (1)

where $f_{LL},f_{LH},f_{HL},f_{HH}$ are the corresponding low-pass and high-pass filters. The specific filters are as follows:


$$f_{LL}=\frac{1}{2}\left[\begin{array}{cc} 1 & 1\\ 1 & 1 \end{array}\right],f_{LH}=\frac{1}{2}\left[\begin{array}{cc} 1 & -1\\ 1 & -1 \end{array}\right],f_{HL}=\frac{1}{2}\left[\begin{array}{cc} 1 & 1\\ -1 & -1 \end{array}\right],f_{HH}=\frac{1}{2}\left[\begin{array}{cc} 1 & -1\\ -1 & 1 \end{array}\right]$$ (2)

These four filters correspond to different frequency information: horizontal, vertical, diagonal, and overall. Wavelet transform convolution (WTConv) effectively extracts multi-scale features in the frequency domain by combining wavelet transformation with convolution operations. The core principle is to decompose the input into different frequency band information, perform convolution operations on these bands, and finally reconstruct the output containing more feature information through inverse wavelet transformation. This convolution method effectively expands the receptive field and enhances the model’s feature extraction capabilities in complex scenes.

**Fig 5:**
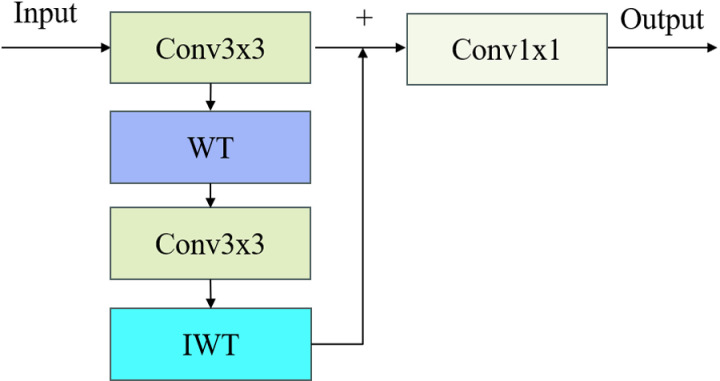
Structure of WSDConv.

In this paper, we attempt to integrate wavelet transform convolution into depthwise separable convolution, forming a novel convolution module called WSDConv. The structure of this convolution block is illustrated in Fig 5:

By utilizing multi-frequency decomposition through wavelet transformation, this convolution block significantly enhances the model’s receptive field and its ability to capture global information while maintaining computational efficiency. Furthermore, WSDConv enhances the model’s capability to extract edge and detail features, leading to excellent performance in complex backgrounds, small target detection, and noise resistance. We replaced some of the basic convolution blocks in the model’s backbone network with WSDConv, and the improved model achieved excellent performance in experiments for identifying explosive materials at blasting sites.

### Improved cross stage partial structure

In the YOLOv8 backbone network, the C2f (Cross Stage Partial Networks with Focus) module primarily functions to effectively extract and fuse multi-scale features through partial feature cross-layer connections and residual structures, thereby enhancing the model’s feature representation capability and detection accuracy. However, challenges arise when handling complex backgrounds and multi-scale targets, including insufficient feature expression, waste of computational resources, inaccurate target localization, limited generalization ability, and loss of important feature information.

**Fig 6:**
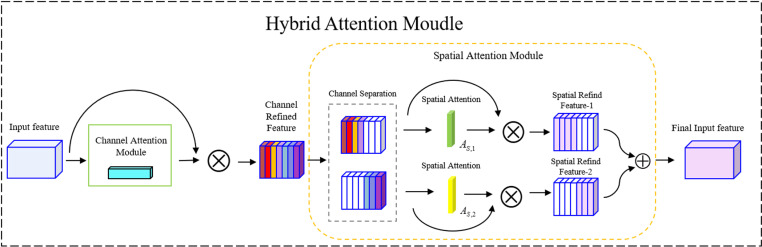
Structure of the channel attention module.

To address these issues, we introduce multi-scale convolutions and parallel attention mechanisms within the C2f module. This approach alleviates the problem of mismatched detector and receptive field sizes caused by downsampling, enhancing the model’s ability to detect multi-scale targets. The structure of the hierarchical attention module is illustrated in Fig 6 [25].

Compared to traditional channel attention modules (ECA) and Convolution Block Attention Modules (CBAM) [26], this structure consists mainly of two sub-modules: the channel module and the spatial module. The channel attention module incorporates an adaptive pooling mechanism to improve feature information extraction, while the spatial attention module employs channel separation techniques to reallocate the weights of the generated channel attention map, enabling the acquisition of richer feature information.

#### Channel attention module.

**Fig 7:**
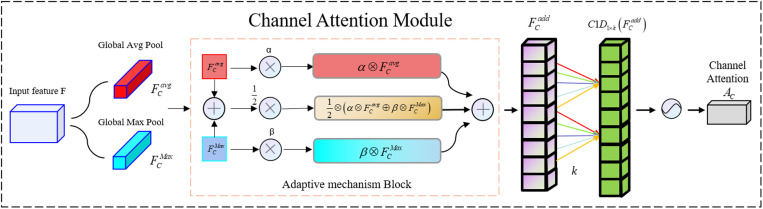
Structure of the Channel Attention Module.

The channel attention module leverages the characteristics of max pooling and average pooling, incorporating an adaptive mechanism during the feature extraction process of both pooling operations. Additionally, a 1D convolution is used to decrease the model’s computational burden while boosting its capability to fuse global feature information. The detailed steps are illustrated in Fig 7.

The global information and prominent feature details of the intermediate feature maps are extracted via average pooling and max pooling, respectively, generating the average-pooled feature $F_{avg}$ and the max-pooled feature $F_{\max}$. These two different feature tensors are then combined through an adaptive mechanism to produce more enriched features $F_{add}\in R^{1\times 1\times C}$. In the adaptive mechanism, two parameters, *α* and *β*, are learned through backpropagation and optimizers. The adaptive mechanism process is represented by Eq 3, where  ⊗  denotes element-wise multiplication, and  ⊕  represents element-wise addition:


$$F_{C}^{add}=\frac{1}{2}⊗\left(F_{C}^{avg}⊕F_{C}^{\max}\right)⊕\alpha ⊗F_{C}^{avg}⊕\beta ⊗F_{C}^{\max}$$ (3)

Both *α* and *β* are initialized as floating-point numbers with a value of 0.5. These two trainable parameters create an adaptive mechanism between the two types of pooled features during the feature extraction process. Additionally, in order to better capture complex relationships between different channels, we perform fast one-dimensional convolution. This approach not only allows for better fusion of information across channels but also ensures that the feature map’s dimensionality is unaffected.

The size of the one-dimensional convolution kernel is set to *k*, representing the interaction between *k* neighboring elements. This is explained in detail in the ECA-Net paper [27] by Eq 4:


$$k=\phi \left(C\right)={\left|t\right|}_{odd}={\left|\frac{\log_{2}^{\left(C\right)}}{\gamma }+\frac{b}{\gamma }\right|}_{odd}$$ (4)

The value of *k* is based on the number of channels *C*, along with two hyperparameters *b* and *γ*, which are typically set to 2 and 1, respectively, in this paper. ${\left|t\right|}_{odd}$ represents the downward rounding of *t*, ensuring that it remains an odd number. A sigmoid function activates the output feature tensor, resulting in the feature mapping $F_{add}\in R^{1\times 1\times C}$. The entire operation of the channel attention module is summarized in Eq 5.


$$\begin{array}{l} A_{C}\left(F\right)=\sigma \left(C1D_{1\times k}\left(\frac{1}{2}⊗\left(AvgPool\left(F\right)⊕MaxPool\left(F\right)\right)⊕\alpha ⊗AvgPool\left(F\right)⊕\beta ⊗MaxPool\left(F\right)\right)\right)\\ \begin{array}{cc} & =\sigma \left(C1D_{1\times k}\left(\frac{1}{2}⊗\left(F_{C}^{avg}⊕F_{C}^{\max}\right)⊕\alpha ⊗F_{C}^{avg}⊕\beta ⊗F_{C}^{\max}\right)\right) \end{array}\\ \begin{array}{cc} & = \end{array}\sigma \left(C1D_{1\times k}\left(F_{C}^{add}\right)\right) \end{array}$$ (5)

Where *σ* denotes the sigmoid function and $C1D_{1\times k}$ stands for a one-dimensional convolution with a kernel size of *k*. The kernel size matches that of the sigmoid function.

#### Spatial attention module.

**Fig 8:**
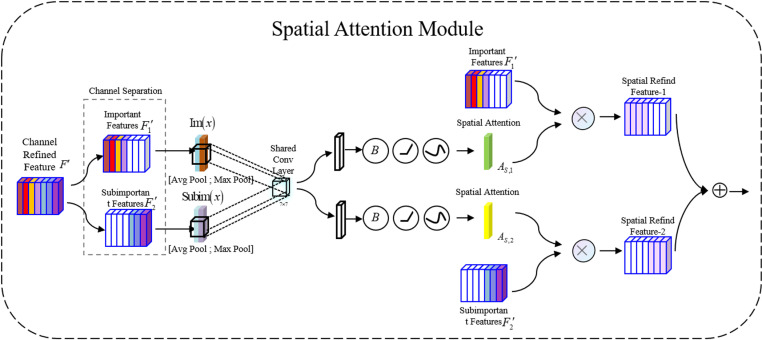
The structure of the spatial attention module.


The weights are assigned according to the channel attention map in the image, dividing the feature map channels into important and less important channels, as shown in Fig 8. In this channel division technique, a hyperparameter *λ* is introduced, which represents the boundary for dividing the channels. The following relationship is established as Eq 6:

$$C_{im}={\left|C_{C-R}\cdot \lambda \right|}_{even}$$ (6)

Here, ${\left|t\right|}_{even}$ represents the nearest even number to *t*, and $C_{C-R}$ represents the channel dimensionality of the refined feature *F*^′^. Typically, the dimensionality of the input channel is an even number, denoted as $C_{im}$. $C_{im}$ can be used to determine the top *n* channels with the highest corresponding values. After determining the value of $C_{im}$, the channel attention map can be divided into important and less important parts. Additionally, the important and less important codes are defined separately. These two codes are then combined with the refined channel features $F_{1}^{\prime }$ and the less important channel feature $F_{2}^{\prime }$.

The channel features $F_{1}^{\prime }$ and $F_{2}^{\prime }$ undergo max pooling and average pooling, respectively, and the results are concatenated to form the input feature maps Imx and $Subim_{\left(x\right)}$ for both channels. Then, convolution operations and normalization are performed to generate the spatial attention maps $A_{S,1}\in R^{H\times W\times 1}$ and $A_{S,2}\in R^{H\times W\times 1}$. The entire spatial attention mechanism calculation process is as shown in Eq 7.


$$\begin{array}{l} A_{S,1}\left(F^{\prime }\right)=\phi \left(C2D_{7\times 7}\left(\left[AvgPool\left(F_{1}^{\prime }\right);MaxPool\left(F_{1}^{\prime }\right)\right]\right)\right)\\ \begin{array}{cc} & = \end{array}\phi \left(C2D_{7\times 7}\left(\left[F_{S,1}^{avg}\right];\left[F_{S,1}^{\max}\right]\right)\right)\\ A_{S,2}\left(F^{\prime }\right)=\phi \left(C2D_{7\times 7}\left(\left[AvgPool\left(F_{2}^{\prime }\right);MaxPool\left(F_{2}^{\prime }\right)\right]\right)\right)\\ \begin{array}{cc} & = \end{array}\phi \left(C2D_{7\times 7}\left(\left[F_{S,2}^{avg}\right];\left[F_{S,2}^{\max}\right]\right)\right) \end{array}$$ (7)

Here, *ϕ* represents the nonlinear normalization and activation operation. $F_{S,i}^{\max}\in R^{H\times W\times 1}$ and $F_{S,i}^{avg}\in R^{H\times W\times 1}$ respectively represent the channel features after max pooling and average pooling operations on $F_{i}^{\prime }$.

**Fig 9:**
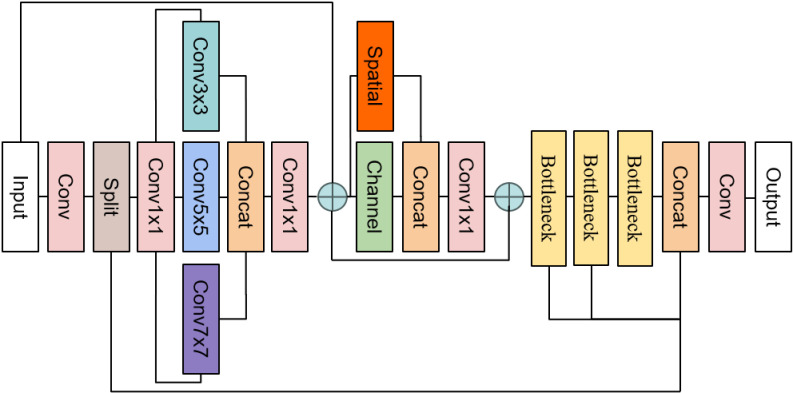
Structure of C2f-MM.

We parallel-link the channel and spatial attention from the hierarchical attention mechanism and incorporate multi-scale convolutions into the C2f module of the neck network, resulting in the C2f-MM module. The structure is depicted in Fig 9.

In this paper, we replace the C2f module in the neck network with the C2f-MM module, which assigns higher weights to more relevant features and spatial details in the image through adaptive mechanisms and channel separation techniques. This design significantly aids in the detection of explosive materials at blasting sites, effectively addressing detection efficiency issues caused by background noise and low image resolution, while still maintaining high detection performance in complex environments. Additionally, this combination of multi-scale convolutions enhances the model’s ability to detect multi-scale targets, providing theoretical support for the detection of small detonators in blasting scenarios.

### Improved loss function

The main purpose of a loss function is to measure the difference between the model’s predictions and the true values, serving as a metric to evaluate the model’s performance. The original loss function of YOLOv8 is primarily based on Intersection over Union (IoU), but it exhibits several shortcomings when dealing with complex backgrounds and multi-scale target detection. These include neglecting differences in the shape and position of bounding boxes, insufficient penalties for out-of-bounds information, and a lack of sensitivity to small targets. In the detection of explosive materials at blasting sites, the varying orientations and significant aspect ratio differences of detonator leads render the IoU loss function ineffective. Thus, we replace the original loss function with WIoU (Wise-IoU) to effectively tackle these issues.

**Fig 10:**
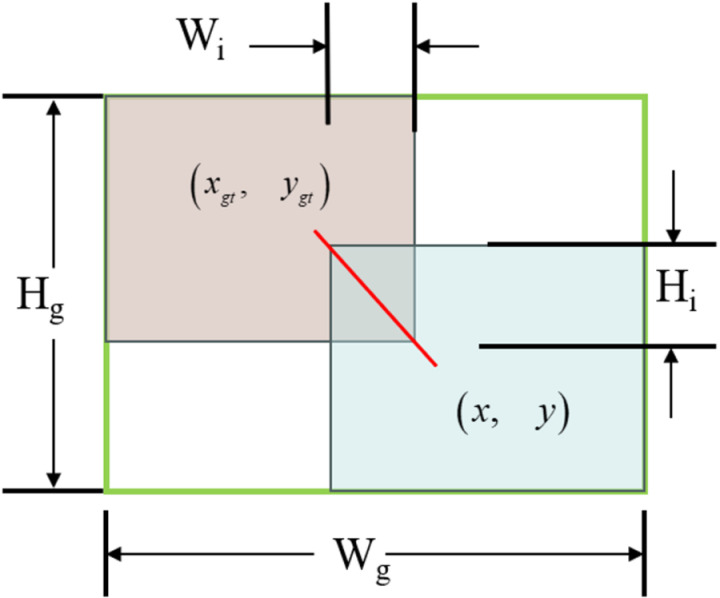
Overlap between predicted box and ground truth box.

In this paper, we specifically adopt Wise-IoU, which introduces a weighting mechanism for bounding box aspect ratios and shapes, allowing for better adaptation to the irregular, elongated objects represented by detonators, thereby enhancing detection accuracy. Let $\vec{B}=\left[\begin{array}{cccc} x & y & w & h \end{array}\right]$ denote the anchor box and B→gt=xgtygtwgthgt denote the target box, with detailed parameters illustrated in Fig 10:

The definition of the WIoU loss function is as follows:


$$L_{WIoUv1}=R_{WIoU}L_{IoU}$$ (8)

$$L_{IoU}=1-IoU=1-\frac{W_{i}H_{i}}{S_{u}}$$ (9)

$$R_{WIoU}=\exp\left(\frac{{\left(x-x_{gt}\right)}^{2}+{\left(y-y_{gt}\right)}^{2}}{{\left(W_{g}^{2}+H_{g}^{2}\right)}^{*}}\right)$$ (10)

where: $R_{WIoU}\in [\left.1,e\right)$, $L_{IoU}\in \left[0,1\right]$. The superscript * indicates that the term has been detached from the computational graph.

WIoU incorporates weighted considerations for the distances, shapes, and sizes of bounding boxes, thereby enhancing the model’s localization ability in complex scenarios. Additionally, it provides richer gradient information, technically supporting the model in detecting explosive materials at blasting sites. This results in a more stable training process and improved overall detection accuracy.

## Experimental results

### Dataset description

**Fig 11:**
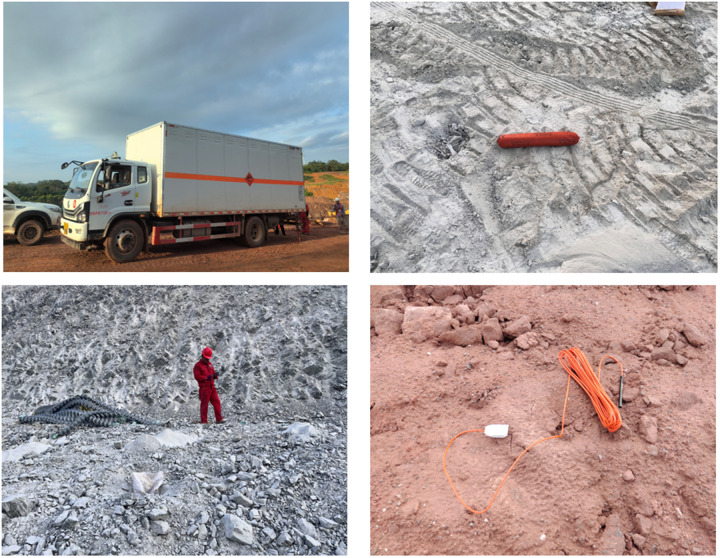
Four different categories in the dataset: explosives, detonators, explosive material vehicles, and personnel.

This study assembled a dataset comprising 11,200 blasting site image samples, encompassing three categories of explosive materials and one essential detection category: detonators, explosives, explosive vehicles, and personnel. Among these, there are 1,200 detonator samples that represent various morphologies of detonators encountered in actual blasting operations. The images were annotated using the labeling tool LabelImg, and the annotation results were converted into a text format, including object IDs, positional coordinates, and width/height information. Subsequently, the dataset was partitioned into training, validation, and testing sets in an 8:1:1 ratio. A schematic of the dataset is presented in Fig 11.

In addition, the other experimental datasets used in this paper are the PASCAL Visual Object Classes (VOC) 2012 dataset [28] (open access link https://host.robots.ox.ac.uk/pascal/VOC/voc2012) and the COCO128 dataset [29] (open access link https://github.com/ultralytics/yolov5).

We validated the category distribution of our custom-built dataset using the Chi-square test based on the actual distribution of categories within the dataset. The results indicated a balanced category distribution. However, regarding the distribution of target sizes, the Chi-square test revealed a significant imbalance (Chi-square statistic: 493.0, p-value: 3.17e-109). To address the issue of imbalanced target sizes, we employed various data augmentation techniques during training, including Mosaic and Mixup augmentations, Hue, Saturation, and Value (HSV) adjustments, random scaling and translation, and horizontal flipping. These techniques aim to enhance the model’s ability to detect targets of varying sizes. Furthermore, to further demonstrate the scientific validity of our custom-built dataset, we conducted a comparative analysis with the public datasets COCO128 and VOC2012. The specific results are presented in Table 1.

**Table 1:** Statistical analysis of different datasets.

Dataset	Number of Categories	Chi-square Statistic	P-value
**Mydata**	4	493.0	3.17e-109
**COCO128**	80	941.0	1.2e-206
**VOC2012**	20	27,450.0	0

VOC 2012 encompasses 20 categories, COCO128 includes 80 categories, while our custom-built dataset comprises only 4 categories. Despite the smaller number of categories, the dataset is highly targeted, specifically designed for object detection in blasting sites, thereby enhancing detection accuracy for specific tasks. We validated the category distribution balance of the custom-built dataset, VOC2012, and COCO128 using the Chi-square test. The results indicate that the category distributions of all three datasets do not exhibit significant differences, suggesting that all are suitable for object detection tasks and possess similar characteristics in terms of category balance.

Regarding target size distribution, although the custom-built dataset, VOC2012, and COCO128 all exhibit significant imbalances (with extremely large Chi-square statistics and negligible p-values), the size distribution of the custom-built dataset is more concentrated (e.g., targets in blasting sites are typically of medium to small sizes). This concentration confers a distinct advantage for the dataset’s application in specific tasks such as explosive detection and blasting site monitoring. In contrast, VOC2012 and COCO128 encompass a broader range of target sizes, resulting in lower adaptability for specialized tasks. Therefore, it is evident that while the custom-built dataset is similar to public datasets in category distribution, it offers greater specificity in target size adaptability, thereby better catering to the demands of object detection tasks in blasting site environments.

### Network configuration and parameter tuning

This study was conducted on the Windows 11 operating system, utilizing Python 3.8.0 and trained under the PyTorch 1.12.1 deep learning framework. During the training process, an NVIDIA GeForce GTX 1070 GPU was employed. To optimize model performance, we adopted the Stochastic Gradient Descent (SGD) optimizer with a total of 200 training epochs and a batch size of 16. The initial learning rate was set to 0.01, with a momentum parameter of 0.9 and a weight decay of 0.0005. The specific hyperparameter configurations and tuning details are presented in Table 2.

**Table 2:** Hyperparameter configuration and tuning details.

Hyperparameter	Value	Description
**Operating System**	Windows 11	System configuration
**Programming Language**	Python 3.8.0	Programming language used
**Deep Learning Framework**	Pytorch 1.12.1	Deep learning framework utilized
**GPU Model**	GeF GTX 1070	GPU model used for training
**Optimizer**	SGD	Stochastic Gradient Descent optimizer employed
**Number of Epochs**	200	Total number of training epochs
**Batch Size**	16	Number of training samples per batch
**Initial Learning Rate**	0.01%	Initial learning rate (0.01% corresponds to 0.0001)
**Momentum**	0.9	Momentum parameter for the optimizer
**Weight Decay**	0.0005	Weight decay coefficient
**Data Augmentation**	True	Enable data augmentation
**Mosaic**	1.0	Probability of applying Mosaic augmentation (1 = enabled, 0 = disabled)
**Mixup**	0.1	Probability of applying Mixup augmentation (0.1 = enabled)
**HSV Hue**	0.015	Hue variation range, representing the maximum hue change
**HSV Saturation**	0.7	Saturation variation range, representing the maximum saturation change
**HSV Value**	0.4	Brightness variation range, representing the maximum brightness change

Furthermore, regarding the hyperparameter tuning process, the setting of the learning rate was adjusted based on preliminary experimental results and optimized through multiple validation experiments to enhance training performance. The momentum parameter and weight decay were configured based on prior experience, aiming to accelerate the model’s convergence speed and reduce overfitting. These data augmentation technique parameters contribute to improving the model’s generalization capability, particularly in complex scenarios where data augmentation strategies significantly enhance the model’s robustness. Each parameter selection and adjustment was grounded in initial experimental findings and refined through multiple validation optimizations to ensure the model’s superior performance across different training datasets.

### Evaluation criteria

To assess the performance of the proposed model, we employed several metrics, including Recall (R), Precision (P), and Mean Average Precision (mAP), to measure the detection accuracy of explosives at the blasting site. Specifically, R, P, and mAP are defined as follows:


$$\mathrm{Pr}ecision=\frac{TP}{TP+FP}$$ (11)

Recall=TPTP+FN (12)

$$mAP=\frac{\sum_{i=1}^{N}AP_{i}}{N}$$ (13)

In this context, TP represents the number of target explosives accurately detected by the model, FP refers to the explosives that were incorrectly predicted, and FN indicates the number of explosives missed by the model. *N* stands for the number of categories, while AP denotes the average precision for a specific detection category, defined as follows:


$$AP=\sum_{i=1}^{n-1}\left(r_{i+1}-r_{i}\right)p_{\mathrm{int}er}\left(r_{i}+1\right)$$ (14)

The presented formula first arranges all predicted bounding boxes in descending order by their confidence levels. Following this, the precision at each position is computed. Eventually, these precision scores are weighted and averaged to determine the mean average precision (mAP). In the formula, *n* denotes the total number of predicted boxes, while $r_{i}$ indicates the number of true positives within the top *i* predicted boxes sorted by confidence. $p_{\mathrm{int}er}$ refers to the Intersection-over-Union (IOU) between the predicted box and the actual ground truth. Generally, a threshold is applied to $p_{\mathrm{int}er}$, and if the IOU between the predicted and ground truth boxes surpasses this threshold, the prediction is deemed a successful detection.

The formula ranks all predicted bounding boxes based on their confidence levels, from highest to lowest, and then calculates the precision at each ranking. These precision scores are subsequently weighted and averaged to compute the mean average precision (mAP). In the formula, *n* stands for the total predicted boxes, and $r_{i}$ indicates the true positives in the top *i* predictions sorted by confidence. $p_{\mathrm{int}er}$ refers to the intersection-over-union (IOU) between the predicted and actual boxes. Generally, a threshold is established for $p_{\mathrm{int}er}$, and if the IOU exceeds this limit, the detection is marked as correct.

### Training results analysis

**Fig 12:**
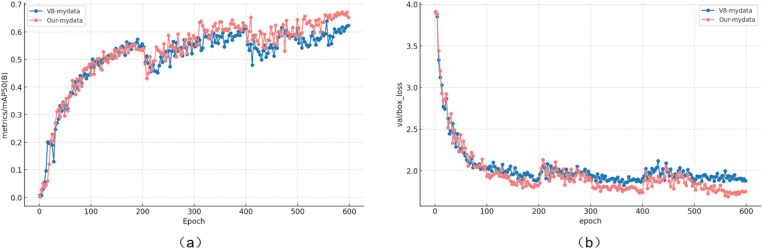
The training results of WA-YOLO and YOLOv8 on the custom blasting site dataset are presented, where (a) shows the mAP_0.5_ comparison, and (b) illustrates the box loss training comparison.

To boost the model’s generalization and training stability, we conducted three independent training sessions, each lasting 200 epochs. After each session, we saved the model weights that achieved the best performance on the validation set and loaded these optimal weights for the next training round. The trends for mAP_0.5_ versus epochs and box-loss versus epochs are shown in Fig 12.

Furthermore, to thoroughly validate the detection efficiency of the WA-YOLO model on the custom-built dataset, this study assessed the performance of explosive material detection in blasting sites using multiple metrics, including Precision, Recall, and Mean Average Precision (mAP). Table 3 presents the detection results for each category of the custom-built dataset using both WA-YOLO and the standard YOLOv8 models. The results in the table indicate that the improved WA-YOLOv8 model outperforms the baseline YOLOv8 model across multiple key metrics.

**Table 3:** Detection results of WA-YOLO and YOLOv8 on the custom-built dataset.

	YOLOv8			WA-YOLO		
**Class**	Precision	Recall	mAP_0.5_	Precision	Recall	mAP_0.5_
**explosive**	0.738	0.996	0.995	**0.760**	0.996	0.995
**detonator**	0.518	0.368	0.403	**0.547**	0.362	**0.485**
**people**	0.561	0.258	0.316	**0.618**	**0.338**	**0.450**
**Explosive vehicle**	0.448	0.444	0.474	**0.816**	**0.496**	**0.489**
**All**	0.566	0.517	0.547	**0.685**	**0.589**	**0.673**

From the trend analysis in Fig 12, it can be concluded that under the same conditions, our model demonstrates superior generalization ability, with lower validation loss and a slight advantage in the mAP_0.5_ metric. Notably, the improved model performs significantly better during the middle and later stages of training. Furthermore, the data from Table 1 clearly shows that the WA-YOLO algorithm achieves higher values in core metrics such as Recall (R) and mean Average Precision (mAP). In particular, for the detonator detection category, the mAP_0.5_ improves by 8.2%.

To comprehensively compare the detection performance of WA-YOLO, we analyzed the F1-score trend of the improved model alongside the baseline YOLOv8 model at the same threshold. The F1-score is an overall metric that takes into account both Recall and Precision, calculated using the following formula:


F1score=2×Precision×RecallPrecision+Recall (15)

**Fig 13:**
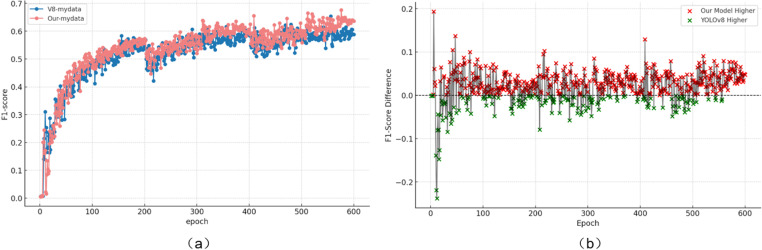
F1 Score Comparison between WA-YOLO and YOLOv8. Where (a) is the F1 score chart, and (b) is the F1 score difference chart.

The experimental results are presented in Fig 13. The comparison shows that the F1-score of both detection models increases with the threshold, eventually stabilizing. However, at the same threshold, the improved WA-YOLO model consistently outperforms the baseline model. The score difference in the table demonstrates that the improved model surpasses the baseline model in most training rounds, indicating that the proposed model achieves a better balance between Recall and Precision.

Furthermore, we performed a cross-dataset analysis to test the robustness of WA-YOLO. The “people” category in the custom-built dataset was mapped to the “person” category in the COCO128 dataset, and the WA-YOLO model trained on the custom-built dataset was directly applied for detection on the COCO128 dataset. Through this cross-dataset testing, we were able to assess the generalization and adaptability of WA-YOLO in different data environments. Specifically, we first completed the training of the WA-YOLO model on the custom-built dataset to ensure that the model had sufficiently learned the features of the “people” category. Then, we mapped the “people” category in the detection results to the “person” category in COCO128, and evaluated the detection performance using COCO evaluation tools (such as pycocotools). The experimental results show that WA-YOLO performs better than the baseline model YOLOv8 on the COCO128 dataset, as shown in Table 4.

**Table 4:** Cross-dataset evaluation: Performance of WA-YOLO and YOLOv8 on the COCO128 dataset.

Models	Recall	mAP_0.5_	mAP_0.75_
**V8**	0.45	0.31	0.29
**WA-YOLO**	**0.51**	**0.44**	**0.36**

The results demonstrate that WA-YOLO exhibits strong robustness and generalization ability across different datasets, maintaining good detection performance under varying data conditions. Furthermore, WA-YOLO excels at higher IoU thresholds (mAP@0.75), indicating a distinct advantage in object localization accuracy. These findings further validate the effectiveness of WA-YOLO in practical applications, particularly in scenarios with diverse data distributions and class mappings, where it continues to deliver stable and efficient detection performance.

### Ablation studies

To validate the specifics of various enhancements made to the model and the effectiveness of the proposed methods, we conducted a series of ablation studies for comparative analysis. Initially, we tested our model on the COCO128 dataset, with detailed experimental results presented in Table 5.

**Table 5:** Model performance evaluation on the COCO128 dataset.

Models	WIoU	WSDConv	C2f-MM	Precision	Recall	mAP_0.5_	mAP_0.5:0.95_
**V8**				0.578	0.306	0.341	0.213
**A**	√			0.525	**0.369**	**0.344**	**0.285**
**B**		√		**0.710**	**0.455**	**0.554**	**0.320**
**C**			√	**0.583**	0.288	**0.346**	0.212
**WA-v8**	√	√	√	**0.612**	**0.386**	**0.449**	**0.265**

The data in Table 5 indicates that the improved model shows enhancements across various metrics. Group A results reveal that incorporating the WIoU loss function improves Recall, mAP0.5, and mAP0.5:0.95, despite a slight drop in Precision. This suggests that the WIoU loss function can enhance model efficiency in specific areas.

Group B and Group C correspond to the results obtained after introducing WSDConv and C2f-MM, respectively. Both experiments show improvements in performance, with Group B results being particularly notable, indicating that the introduction of WSDConv significantly enhances the model’s performance. However, due to the limited scale of the COCO128 dataset, the experimental results may not be convincing. Therefore, we conducted the same comparative experiments on the VOC2012 dataset, with the results presented in Table 6:

**Table 6:** Model performance comparison on the VOC2012 dataset.

Models	WIoU	WSDConv	C2f-MM	Precision	Recall	mAP_0.5_	mAP_0.5:0.95_
**V8**				0.671	0.563	0.611	0.398
**A**	√			**0.689**	0.557	**0.612**	**0.412**
**B**		√		**0.679**	**0.570**	**0.625**	**0.412**
**C**			√	**0.678**	0.549	0.601	**0.399**
**WA-v8**	√	√	√	**0.678**	**0.576**	**0.627**	**0.425**

From the data in Table 6, it can be concluded that the improved model also achieves significant improvement on the VOC2012 dataset. The results of Group A experiments demonstrate that when the WIoU loss function is introduced, the model shows an improvement in Precision, mAP_0.5_, and mAP_0.5:0.95_, indicating that enhancing the accuracy of the calculation of bounding box overlap effectively improves model accuracy, providing support for subsequent experiments. In Group B experiments, WSDConv also shows notable improvement, surpassing the baseline model across all metrics, suggesting that the introduction of WSDConv significantly enhances the model’s feature extraction capabilities, providing experimental evidence for detecting pyrotechnics in complex backgrounds. In Group C experiments, although there is a slight decline in Recall and mAP_0.5_ after introducing C2f-MM, the model outperforms the baseline across all other metrics.

From the above model comparison experiments, it can be concluded that the main enhancements in model improvement have demonstrated substantial effectiveness on real-world datasets, indicating the feasibility of such improvements. Furthermore, we conducted the same ablation experiments on our custom-built dataset for pyrotechnics detection at blast sites to address our specific problem, with the results shown in Table 7.

**Table 7:** Ablation experiments on custom dataset.

Models	WIoU	WSDConv	C2f-MM	Precision	Recall	mAP_0.5_	mAP_0.5:0.95_
**YOLOv8**				0.566	0.517	0.547	0.388
**A**	√			**0.582**	0.499	**0.553**	0.355
**B**		√		0.535	**0.522**	0.545	0.358
**C**			√	**0.665**	**0.534**	**0.581**	**0.389**
**WA-YOLO**	√	√	√	**0.685**	**0.589**	**0.673**	**0.462**

From the data in the table, it can be concluded that our model shows significant improvements on the pyrotechnics dataset from the blast site, with different module integrations leading to varying performance enhancements. Group A experiments indicate that the introduction of the WIoU loss function increases Precision and mAP_0.5_ by approximately 2%. The results from Group B experiments demonstrate that on the pyrotechnics dataset, the introduction of WSDConv effectively compensates for the decline in Recall caused by the WIoU loss function. In Group C experiments, all metrics show significant improvements, with Precision increasing by 10% and mAP_0.5_ increasing by 3.4%, suggesting that the introduction of multi-scale convolution and parallel attention mechanisms is highly effective for pyrotechnics detection in blast sites.

Additionally, to comprehensively evaluate the impact of each component on the model, we conducted measurements of frames per second (FPS), parameter count, and floating point operations (GFLOPs) on our custom dataset. This was done by systematically removing or replacing specific components in the model to assess their contribution to overall performance. The experimental results are presented in Table 8.

**Table 8:** Module ablation study.

Models	WIoU	WSDConv	C2f-MM	FPS	Params/10^6^	GFLOPs
**V8**				30	30	8.1
**A**	√			30	30.3	8.1
**B**		√		**33**	**26**	**7.6**
**C**			√	28	33	13.5
**WA-YOLO**	√	√	√	**32**	**28**	12

The experimental results in Tables 7 and 8 indicate that, compared to the baseline model, the introduction of the WSDConv module leads to an improvement in FPS to 33, a reduction in parameter count to 26 million, and a decrease in GFLOPs to 7.6. This highlights the effectiveness of the WSDConv module in reducing computational complexity and accelerating inference speed through the incorporation of wavelet transforms, although it slightly compromises accuracy. Additionally, the inclusion of the attention mechanism undoubtedly increases the model’s complexity, but it brings a notable improvement in accuracy. WA-YOLO, by integrating these modules, leverages their individual strengths, achieving an optimal balance between speed and accuracy, and performs exceptionally well in complex scenarios.

In summary, we conducted ablation experiments on the WA-YOLO and YOLOv8 models across three datasets, verifying the impact of each enhancement on model performance. The results highlight the robustness of the improved model in different scenarios, demonstrating its generalization capability and providing evidence for its practical application in this study.

### Detection visualization results comparison

**Fig 14:**
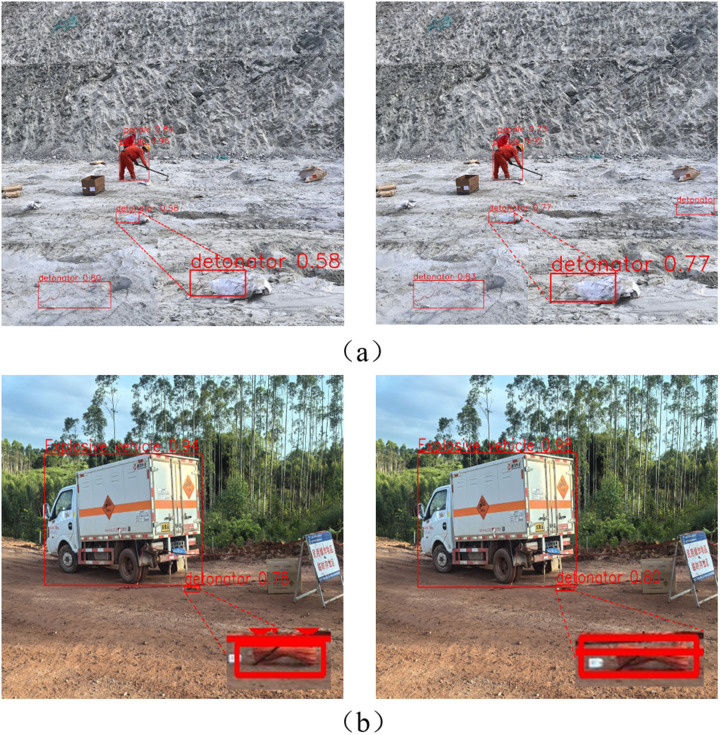
Compares the detection performance of the baseline model and the improved model in actual blasting scenarios. The left side shows the detection results of the YOLOv8 model, while the right side shows the detection results of the improved WA-YOLO model.

To more comprehensively evaluate the detection performance of the improved model, this paper tested firework products on site in various blasting work environments. The detection results are shown in Fig 14. By comparing the experimental results, it was found that the baseline model performed significantly inadequately in complex backgrounds, such as obstruction by debris and detection of small-diameter pipes. In these complex environments, the baseline model struggled to effectively distinguish between the target and the background, leading to a significant decrease in detection accuracy and the risk of serious errors such as missed detections. As a result, the baseline model failed to meet reasonable expectations for accuracy and reliability. In contrast, the improved model demonstrated significantly higher detection accuracy and reliability in these environments.

**Fig 15:**
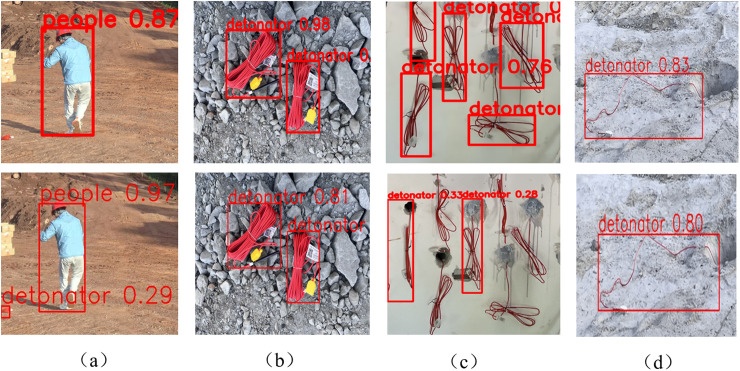
Shows the detonator detection results of the improved model in different scenarios at the blasting site.

To solve the detection challenge posed by the varying postures of detonator lead wires before and after use at blasting sites, this study detects detonators with various open and closed states in the field to verify the accuracy of the improved model. The detection results are shown in Fig 15, where the first row displays the results of WA-YOLO and the second row shows the results of the baseline model. In subfigure (a), WA-YOLO detects a detonator dropped in a wide area with 29% confidence. Additionally, for the case where the detonator’s lead wires are fully extended, WA-YOLO still achieves 83% confidence, which is 3 percentage points higher than the baseline model. In subfigure (c), where the lead wires are not fully extended during blast dismantling, the improved model shows a significant performance improvement. This fully demonstrates that WA-YOLO can adapt to most situations at blasting sites, meeting reasonable expectations for accuracy and reliability.

**Fig 16:**
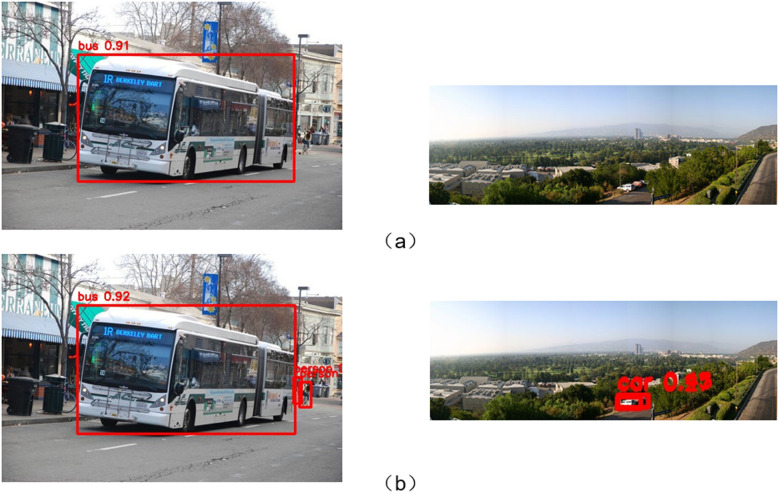
Comparison between the WA-YOLO model and the YOLOv8 model on public datasets. (a) shows the detection results of the YOLOv8 model, and (b) shows the results of the WA-YOLO model.

Additionally, we performed detection visualizations on the VOC2012 and COCO128 datasets, with the results shown in Fig 16. From the comparison in the left-side image, it can be observed that the improved model is able to detect the “person” target that the baseline model missed. The detection accuracy for large-scale targets like “bus” is also higher with the improved model compared to the baseline, indicating that our model performs better in detecting targets of varying scales. From the comparison in the right-side image, it can be seen that the improved model is better at detecting the “car” target in complex backgrounds, demonstrating its superiority in small object detection tasks. In summary, the experimental results show that our improved model has excellent detection performance in tasks involving complex backgrounds and multi-scale target detection.

### Comparison of detection results from multiple models

To further evaluate the performance of the WA-YOLO model, this study conducted a comprehensive comparison with three single-object detection models (YOLOv5, YOLOv7 [30], YOLOX [31]) and two two-object detection models (Faster R-CNN [32], Mask R-CNN [33]). The detailed experimental results on the custom dataset are presented in Table 9. Additionally, comparisons were made on the COCO128 and VOC2012 public datasets, with experimental results shown in Table 10 (COCO128 dataset) and Table 11 (VOC2012 dataset), respectively.

**Table 10:** Comparison of detection results of mainstream models on the COCO128 dataset.

Model	Params/10^6^	GFLOPs	mAP_0.5:0.95_%
**YOLOv5n**	16	4.1	20.8
**YOLOv7**	43	15.	20.4
**YOLOX**	9.	54.	23.2
**Faster R-CNN**	13	–	24.1
**Mask R-CNN**	42	–	25.7
**YOLOv8**	31	8.7	21.3
**WA-YOLO**	**30**	**12.**	**26.5**

**Table 11:** Comparison of detection results of mainstream models on the VOC2012 dataset.

Model	Precision	mAP_0.5_%	mAP_0.5:0.95_%
**YOLOv5n**	0.645	59.1	38.1
**YOLOv7**	0.631	61.3	39.4
**YOLOX**	0.64	61.2	39.2
**Faster R-CNN**	0.523	64.2	34.1
**Mask R-CNN**	0.521	60.1	33.7
**YOLOv8**	0.671	61.1	39.8
**WA-YOLO**	**0.678**	**0.627**	**0.425**

**Table 9:** Comparison of detection results of mainstream models on the custom dataset.

Model	Params/10^6^	Training Time/h	mAP_0.5_%
**YOLOv5n**	17	6.5	64.3
**YOLOv7**	46	12	66.5
**YOLOX**	9.	5.8	66.1
**Faster R-CNN**	13	14	61.5
**Mask R-CNN**	42	16	62.2
**YOLOv8**	30	8.5	54.7
**WA-YOLO**	28	10	**67.3**

In this study, we conducted a detailed performance comparison of mainstream object detection models on three datasets: our self-constructed blasting site dataset (Table 9), the COCO128 dataset (Table 10), and the VOC2012 dataset (Table 11). The results demonstrate that the proposed WA-YOLO model outperforms other mainstream models across multiple key performance metrics, as follows:

On the self-constructed blasting site dataset (Table 9), WA-YOLO achieved a mAP@0.5 of 67.3%, significantly surpassing other popular object detection models, such as YOLOv5n (64.3%), YOLOv7 (66.5%), YOLOX (66.1%), Faster R-CNN (61.5%), Mask R-CNN (62.2%), and YOLOv8 (54.7%). Compared to higher-performance models like YOLOv7 and YOLOX, WA-YOLO achieved further improvements in detection accuracy. In terms of parameter size, WA-YOLO has 28 × 10^6 parameters, which is smaller than YOLOv7 (46 × 10^6) and Mask R-CNN (42 × 10^6), but only slightly larger than the lightweight models YOLOv5n (17 × 10^6) and YOLOX (9 × 10^6). Additionally, WA-YOLO’s training time is 10 hours, which remains within a reasonable range compared to higher-performing models (e.g., YOLOv7 and Mask R-CNN), showcasing excellent efficiency. Overall, WA-YOLO achieves a significant improvement in detection accuracy while maintaining a low computational cost and resource usage. This demonstrates its exceptional adaptability and practicality in complex environments, making it an ideal solution for the detection of explosive items in blasting sites, combining both performance and efficiency.

On the COCO128 dataset (Table 10), WA-YOLO achieved a mAP@0.5:0.95 of 26.5%, outperforming YOLOv5n (20.8%), YOLOv7 (20.4%), YOLOX (23.2%), Faster R-CNN (24.1%), Mask R-CNN (25.7%), and YOLOv8 (21.3%). This result indicates that WA-YOLO exhibits strong generalization ability across different datasets, maintaining high detection performance in diverse tasks. Experimental results on the VOC2012 dataset (Table 7) show that WA-YOLO outperforms other mainstream models across all evaluation metrics, highlighting its superior detection capability. Specifically, WA-YOLO demonstrated significant improvements in precision, mAP@0.5%, and mAP@0.5:0.95%, validating its superior performance on standard datasets. These results further support the effectiveness and robustness of WA-YOLO in diverse and complex scenarios.

In summary, the experimental results on the self-constructed blasting site, COCO128, and VOC2012 datasets show that WA-YOLO outperforms current mainstream object detection models. It exhibits comprehensive advantages in detection accuracy, recall rate, and computational efficiency. WA-YOLO not only demonstrates outstanding performance in complex and diverse detection tasks but also maintains a lightweight and efficient design, making it suitable for edge computing and resource-constrained environments. This highlights its broad potential and significant advantages in practical applications.

## Conclusion

To address the challenges in explosive item detection in blasting sites, this study proposes the WA-YOLO model. The model incorporates wavelet transform convolution and a novel CSP structure that reconstructs the C2f module in the neck network. This modification allows the improved model to filter background noise while enhancing target features and boosting performance in multi-scale object detection. Experimental results demonstrate that the proposed method achieves satisfactory performance on our self-constructed detection dataset, effectively solving the detection challenges posed by the varying wireline patterns of detonators at blasting sites. The model not only accurately identifies detonators but also maintains high detection efficiency. Furthermore, on public datasets, the improved model shows significant optimization, indicating strong generalization ability and robustness.

However, the domain-specific design of WA-YOLO, while significantly enhancing explosive item detection performance, also introduces some trade-offs. First, the introduction of wavelet separable depth convolution (WSDConv) and parallel attention mechanisms strengthens the model’s feature extraction capability but also increases computational complexity, which may impact processing speed in real-time applications. Additionally, while WA-YOLO has been optimized for the complex environment of blasting sites, particularly in handling the varying poses of detonator wirelines and large-scale target differences, this specific optimization could limit the model’s generalization ability in other application scenarios. For different domains or datasets with distinct features, further adjustments and optimizations may be required to maintain high performance. Moreover, although WSDConv reduces the computational parameter size to some extent, the additional complexity introduced during its implementation and optimization increases training time, which could become a bottleneck in large-scale datasets or rapid iteration development environments. Future work will focus on developing more efficient optimization methods, exploring cross-domain transfer learning, and multi-task learning techniques to further improve model performance. WA-YOLO holds promise for applications in blasting safety supervision systems and intelligent blasting models, expanding its application range and practical benefits, and further advancing object detection in blasting safety inspection.

### Integration and practical application considerations

To further understand the practicality of the WA-YOLO model, this study also explores its integration with existing safety systems at blasting sites. WA-YOLO can seamlessly interface with on-site surveillance cameras and alarm systems. By performing real-time object detection and analysis, it provides immediate safety alerts, thereby enhancing overall safety management efficiency and response speed. Specifically, WA-YOLO can collaborate with existing monitoring infrastructure to automate the identification of explosive items and anomaly detection, reducing the workload of manual monitoring while improving the accuracy and timeliness of detections.

### Limitations of the model in practical environments

Although WA-YOLO performs excellently in experimental settings, it may still face some challenges in the complex and dynamic environment of a blasting site. First, factors such as strong lighting changes, smoke interference, and dynamic disturbances may affect the model’s detection accuracy and stability. Additionally, the high-intensity vibrations and noise generated by blasting activities could potentially impact the performance of monitoring equipment, indirectly affecting the operation of WA-YOLO. To address these challenges, future research will focus on optimizing the model’s robustness, enhancing its adaptability in extreme environments, and exploring multi-sensor fusion technologies to further improve the overall system performance and reliability.

By comprehensively considering the integration of WA-YOLO with existing safety systems and the potential limitations in blasting site environments, this study provides valuable insights for practical applications, while also pointing the way forward for future model optimization and system upgrades.

## Supporting information

S1 Table Comparison of model training results. (DOCX)

S2 Table Dataset download. (DOCX)
