# Supplementary material for: WA-YOLO: An explosive material detection algorithm for blasting sites based on YOLOv8
Source: PLoS One. 2025 Apr 22;20(4):e0318172. doi: 10.1371/journal.pone.0318172 (PMC12013926; doi:10.1371/journal.pone.0318172)
Supplement: S1 Table — (DOCX) [file pone.0318172.s001.docx]

**S1 Table** Comparison of Model Training Results

| **epoch** | **WA-YOLO val/box_loss** | **YOLOv8 val/box_loss** | **WA-YOLO F1-score** | **YOLOv8 F1-score** | **WA-YOLO metrics/mAP50(B)** | **YOLOv8 metrics/mAP50(B)** |
| --- | --- | --- | --- | --- | --- | --- |
| **1** | 3.9138 | 3.9054 | 0.00476 | 0.006004 | 0.00213 | 0.00666 |
| **2** | 3.9028 | 3.8898 | 0.004973 | 0.006132 | 0.00259 | 0.00882 |
| **3** | 3.899 | 3.8745 | 0.005304 | 0.006054 | 0.03252 | 0.00773 |
| **4** | 3.878 | 3.8529 | 0.005996 | 0.007462 | 0.02796 | 0.00726 |
| **5** | 3.7628 | 3.9437 | 0.009649 | 0.007957 | 0.01066 | 0.01495 |
| **6** | 3.6021 | 3.6733 | 0.200469 | 0.007363 | 0.04784 | 0.00529 |
| **7** | 3.4401 | 3.3291 | 0.20069 | 0.140093 | 0.05097 | 0.0295 |
| **8** | 3.2436 | 3.2794 | 0.244566 | 0.213896 | 0.01123 | 0.03318 |
| **9** | 3.3398 | 3.5027 | 0.161863 | 0.140067 | 0.03276 | 0.03155 |
| **10** | 3.1993 | 3.1211 | 0.171158 | 0.309864 | 0.03841 | 0.05558 |
| **11** | 2.9561 | 3.0909 | 0.021467 | 0.240191 | 0.02344 | 0.01812 |
| **12** | 2.95 | 2.9924 | 0.015532 | 0.253529 | 0.02194 | 0.07492 |
| **13** | 2.928 | 3.0291 | 0.083817 | 0.164645 | 0.04513 | 0.09622 |
| **14** | 2.9242 | 2.9195 | 0.134201 | 0.178189 | 0.10815 | 0.14386 |
| **15** | 2.9207 | 2.8458 | 0.102851 | 0.1822 | 0.03848 | 0.1409 |
| **16** | 2.8397 | 2.7691 | 0.087695 | 0.235494 | 0.05966 | 0.20062 |
| **17** | 2.949 | 3.0197 | 0.096608 | 0.2232 | 0.05827 | 0.17394 |
| **18** | 3.0278 | 2.684 | 0.195163 | 0.162862 | 0.1466 | 0.11936 |
| **19** | 2.8604 | 2.7425 | 0.240901 | 0.286965 | 0.12037 | 0.19568 |
| **20** | 2.5898 | 2.8681 | 0.238149 | 0.173698 | 0.20788 | 0.15094 |
| **21** | 2.8244 | 2.5911 | 0.224674 | 0.236803 | 0.16754 | 0.1662 |
| **22** | 2.9225 | 2.8671 | 0.209964 | 0.23094 | 0.20421 | 0.19939 |
| **23** | 2.6618 | 2.7338 | 0.201731 | 0.259327 | 0.12893 | 0.12709 |
| **24** | 2.7919 | 2.8236 | 0.215479 | 0.23366 | 0.21131 | 0.23067 |
| **25** | 2.517 | 2.6299 | 0.259627 | 0.218319 | 0.22957 | 0.18957 |
| **26** | 2.4569 | 2.6187 | 0.261413 | 0.216921 | 0.18957 | 0.20882 |
| **27** | 2.6997 | 2.4962 | 0.247998 | 0.267604 | 0.23087 | 0.22126 |
| **28** | 2.5815 | 2.4428 | 0.222536 | 0.238453 | 0.20506 | 0.13002 |
| **29** | 2.6365 | 2.5391 | 0.304221 | 0.355894 | 0.28762 | 0.2793 |
| **30** | 2.4968 | 2.4646 | 0.333894 | 0.332837 | 0.27285 | 0.24917 |
| **31** | 2.6854 | 2.4787 | 0.308927 | 0.289593 | 0.26958 | 0.24612 |
| **32** | 2.7504 | 2.6209 | 0.267233 | 0.351611 | 0.22378 | 0.31827 |
| **33** | 2.4245 | 2.4736 | 0.30844 | 0.301482 | 0.27238 | 0.29607 |
| **34** | 2.331 | 2.5684 | 0.336289 | 0.305462 | 0.3103 | 0.26985 |
| **35** | 2.3914 | 2.5131 | 0.38779 | 0.350802 | 0.31207 | 0.30723 |
| **36** | 2.4803 | 2.2557 | 0.2959 | 0.337398 | 0.23989 | 0.30041 |
| **37** | 2.4893 | 2.4549 | 0.368073 | 0.291512 | 0.31914 | 0.2846 |
| **38** | 2.3866 | 2.3115 | 0.258747 | 0.303879 | 0.20511 | 0.31004 |
| **39** | 2.2942 | 2.3433 | 0.337285 | 0.402974 | 0.29 | 0.32206 |
| **40** | 2.369 | 2.2876 | 0.373911 | 0.324992 | 0.29095 | 0.33287 |
| **41** | 2.3284 | 2.3498 | 0.30459 | 0.360585 | 0.27825 | 0.31674 |
| **42** | 2.3365 | 2.2626 | 0.384801 | 0.280326 | 0.32379 | 0.30188 |
| **43** | 2.392 | 2.4436 | 0.387747 | 0.336279 | 0.3329 | 0.31302 |
| **44** | 2.1877 | 2.354 | 0.324429 | 0.35251 | 0.3052 | 0.33907 |
| **45** | 2.4669 | 2.3861 | 0.382408 | 0.323665 | 0.32254 | 0.31602 |
| **46** | 2.2294 | 2.2324 | 0.406598 | 0.371695 | 0.34146 | 0.3445 |
| **47** | 2.2155 | 2.3934 | 0.419818 | 0.28326 | 0.34248 | 0.24409 |
| **48** | 2.3463 | 2.3675 | 0.413921 | 0.360578 | 0.35693 | 0.34764 |
| **49** | 2.4312 | 2.2663 | 0.359501 | 0.400165 | 0.29578 | 0.31939 |
| **50** | 2.1536 | 2.2565 | 0.43526 | 0.369071 | 0.36946 | 0.32894 |
| **51** | 2.1607 | 2.2196 | 0.424362 | 0.387759 | 0.36661 | 0.35247 |
| **52** | 2.2315 | 2.2718 | 0.413363 | 0.381583 | 0.35747 | 0.33458 |
| **53** | 2.228 | 2.1708 | 0.451493 | 0.381103 | 0.37324 | 0.35796 |
| **54** | 2.2337 | 2.2096 | 0.341807 | 0.376259 | 0.33038 | 0.37954 |
| **55** | 2.3536 | 2.2189 | 0.372972 | 0.370177 | 0.31374 | 0.32137 |
| **56** | 2.2588 | 2.1126 | 0.419527 | 0.414782 | 0.35831 | 0.38934 |
| **57** | 2.3178 | 2.1334 | 0.455221 | 0.40243 | 0.36007 | 0.40179 |
| **58** | 2.2708 | 2.1806 | 0.428864 | 0.405856 | 0.36738 | 0.36485 |
| **59** | 2.3114 | 2.2579 | 0.366771 | 0.369603 | 0.35047 | 0.36722 |
| **60** | 2.0946 | 2.155 | 0.433987 | 0.363747 | 0.38361 | 0.38189 |
| **61** | 2.1951 | 2.1303 | 0.413779 | 0.376867 | 0.3698 | 0.37987 |
| **62** | 2.0896 | 2.1666 | 0.41713 | 0.423615 | 0.38729 | 0.40383 |
| **63** | 2.0606 | 2.1057 | 0.452186 | 0.365303 | 0.39023 | 0.36986 |
| **64** | 2.0569 | 2.1077 | 0.476937 | 0.404594 | 0.42314 | 0.37643 |
| **65** | 2.0858 | 2.1529 | 0.459063 | 0.383176 | 0.40635 | 0.35178 |
| **66** | 2.0967 | 2.1635 | 0.483563 | 0.466742 | 0.41475 | 0.41159 |
| **67** | 2.163 | 2.0909 | 0.474488 | 0.435531 | 0.40499 | 0.403 |
| **68** | 2.126 | 2.0368 | 0.429908 | 0.448169 | 0.37187 | 0.41683 |
| **69** | 2.1723 | 2.0474 | 0.458907 | 0.458659 | 0.3925 | 0.41544 |
| **70** | 2.2178 | 2.1492 | 0.422865 | 0.456766 | 0.37376 | 0.41857 |
| **71** | 2.1849 | 2.2294 | 0.431707 | 0.440027 | 0.39978 | 0.41681 |
| **72** | 2.1108 | 2.1004 | 0.430786 | 0.435549 | 0.39843 | 0.41905 |
| **73** | 2.1781 | 2.038 | 0.439333 | 0.417076 | 0.42856 | 0.39415 |
| **74** | 2.201 | 2.1762 | 0.466219 | 0.387933 | 0.43133 | 0.34773 |
| **75** | 2.0295 | 2.0829 | 0.489318 | 0.38959 | 0.44043 | 0.36154 |
| **76** | 2.0503 | 2.0793 | 0.468931 | 0.440657 | 0.38849 | 0.42544 |
| **77** | 2.13 | 2.1358 | 0.384514 | 0.426644 | 0.36397 | 0.40381 |
| **78** | 2.0586 | 2.0347 | 0.471321 | 0.452101 | 0.43761 | 0.42273 |
| **79** | 2.0431 | 2.063 | 0.477163 | 0.470056 | 0.43046 | 0.44105 |
| **80** | 2.054 | 2.0241 | 0.480168 | 0.428895 | 0.40382 | 0.43748 |
| **81** | 2.1177 | 1.9699 | 0.472861 | 0.4601 | 0.42256 | 0.45897 |
| **82** | 2.0457 | 2.044 | 0.486807 | 0.451393 | 0.4029 | 0.41403 |
| **83** | 1.9498 | 2.0472 | 0.444976 | 0.441315 | 0.4426 | 0.42733 |
| **84** | 2.0036 | 1.9679 | 0.465125 | 0.426908 | 0.43122 | 0.412 |
| **85** | 2.0641 | 2.0802 | 0.504604 | 0.433347 | 0.46034 | 0.44028 |
| **86** | 2.037 | 1.9866 | 0.511679 | 0.471456 | 0.47079 | 0.45709 |
| **87** | 2.0928 | 2.0036 | 0.472498 | 0.452761 | 0.44921 | 0.42514 |
| **88** | 2.0538 | 2.0189 | 0.517096 | 0.475719 | 0.46696 | 0.44734 |
| **89** | 2.0165 | 1.9673 | 0.504214 | 0.421516 | 0.47035 | 0.42749 |
| **90** | 2.0332 | 2.0634 | 0.476521 | 0.470359 | 0.43325 | 0.4575 |
| **91** | 2.1378 | 2.0259 | 0.491463 | 0.450407 | 0.46363 | 0.43085 |
| **92** | 2.054 | 1.9924 | 0.453138 | 0.450933 | 0.44371 | 0.44533 |
| **93** | 2.0763 | 2.0094 | 0.476425 | 0.460078 | 0.4583 | 0.45958 |
| **94** | 2.0118 | 2.0146 | 0.520434 | 0.453727 | 0.47217 | 0.44354 |
| **95** | 2.0542 | 2.1078 | 0.510428 | 0.486616 | 0.45203 | 0.44243 |
| **96** | 2.0787 | 2.0316 | 0.504159 | 0.441681 | 0.46951 | 0.4321 |
| **97** | 2.0511 | 2.0505 | 0.50769 | 0.479366 | 0.48279 | 0.44345 |
| **98** | 2.0286 | 1.9819 | 0.48999 | 0.470856 | 0.46339 | 0.43911 |
| **99** | 2.0072 | 2.0066 | 0.499673 | 0.480679 | 0.48463 | 0.46252 |
| **100** | 1.9327 | 2.0346 | 0.468054 | 0.475705 | 0.4446 | 0.46989 |
| **101** | 2.0465 | 1.969 | 0.475744 | 0.479005 | 0.46731 | 0.48235 |
| **102** | 1.8515 | 1.9948 | 0.496544 | 0.489052 | 0.48072 | 0.46797 |
| **103** | 1.9522 | 2.0193 | 0.494739 | 0.480776 | 0.4883 | 0.50099 |
| **104** | 1.9967 | 2.0081 | 0.464393 | 0.472162 | 0.46921 | 0.48773 |
| **105** | 1.9943 | 2.0893 | 0.479464 | 0.411381 | 0.43532 | 0.43794 |
| **106** | 1.9118 | 2.0526 | 0.465673 | 0.488921 | 0.44676 | 0.47114 |
| **107** | 1.8869 | 1.9706 | 0.472014 | 0.450653 | 0.44905 | 0.46426 |
| **108** | 1.8675 | 2.0671 | 0.482173 | 0.477116 | 0.47211 | 0.43742 |
| **109** | 1.9364 | 1.9949 | 0.509083 | 0.500187 | 0.48304 | 0.49252 |
| **110** | 1.8967 | 1.9531 | 0.525987 | 0.481156 | 0.50827 | 0.49923 |
| **111** | 1.9551 | 2.0771 | 0.502893 | 0.505964 | 0.49483 | 0.49026 |
| **112** | 1.952 | 2.0988 | 0.521196 | 0.498646 | 0.50204 | 0.48955 |
| **113** | 1.9904 | 2.09 | 0.492479 | 0.495005 | 0.48665 | 0.46233 |
| **114** | 1.8292 | 2.0355 | 0.535462 | 0.471904 | 0.49681 | 0.50294 |
| **115** | 1.9684 | 2.0472 | 0.529558 | 0.516597 | 0.50239 | 0.47902 |
| **116** | 1.9206 | 1.9761 | 0.504765 | 0.499456 | 0.49332 | 0.50267 |
| **117** | 1.884 | 2.0509 | 0.474075 | 0.494108 | 0.45415 | 0.49437 |
| **118** | 1.9738 | 1.9701 | 0.494682 | 0.48049 | 0.46826 | 0.49837 |
| **119** | 1.9273 | 1.9774 | 0.516198 | 0.444672 | 0.48157 | 0.46632 |
| **120** | 1.9194 | 2.0732 | 0.541426 | 0.484248 | 0.50276 | 0.49109 |
| **121** | 1.9852 | 2.0255 | 0.501464 | 0.505417 | 0.46162 | 0.48758 |
| **122** | 1.902 | 2.0387 | 0.488501 | 0.536771 | 0.48808 | 0.52052 |
| **123** | 1.9107 | 2.0062 | 0.518084 | 0.51654 | 0.50624 | 0.54029 |
| **124** | 1.9379 | 2.0126 | 0.516028 | 0.502458 | 0.52782 | 0.50218 |
| **125** | 1.9028 | 2.0741 | 0.526719 | 0.46196 | 0.50156 | 0.47546 |
| **126** | 1.9156 | 2.0905 | 0.531301 | 0.501526 | 0.49912 | 0.48415 |
| **127** | 2.0186 | 1.9797 | 0.525345 | 0.521154 | 0.49453 | 0.50412 |
| **128** | 1.9404 | 1.9926 | 0.509579 | 0.491804 | 0.51115 | 0.51662 |
| **129** | 1.8999 | 1.9469 | 0.502558 | 0.508572 | 0.50999 | 0.52118 |
| **130** | 1.9301 | 2.0743 | 0.524894 | 0.507628 | 0.51034 | 0.49817 |
| **131** | 1.9145 | 2.0617 | 0.498102 | 0.486985 | 0.48743 | 0.48844 |
| **132** | 1.9481 | 2.0677 | 0.524796 | 0.523113 | 0.50846 | 0.514 |
| **133** | 1.9227 | 1.9704 | 0.514853 | 0.500933 | 0.48932 | 0.51251 |
| **134** | 1.8793 | 1.9651 | 0.516289 | 0.501979 | 0.50364 | 0.52559 |
| **135** | 1.854 | 1.9629 | 0.511646 | 0.496701 | 0.47816 | 0.49176 |
| **136** | 1.9546 | 1.9918 | 0.51774 | 0.497494 | 0.50392 | 0.51439 |
| **137** | 1.9243 | 1.9503 | 0.530031 | 0.496775 | 0.51519 | 0.5086 |
| **138** | 1.9952 | 2.028 | 0.538055 | 0.522711 | 0.50555 | 0.52252 |
| **139** | 1.909 | 1.9806 | 0.539646 | 0.526541 | 0.51754 | 0.51841 |
| **140** | 1.9189 | 1.9989 | 0.52752 | 0.478014 | 0.49548 | 0.4859 |
| **141** | 1.8042 | 2.03 | 0.545018 | 0.491848 | 0.52736 | 0.5115 |
| **142** | 1.9342 | 2.0169 | 0.53937 | 0.497406 | 0.50624 | 0.50357 |
| **143** | 1.8719 | 1.9737 | 0.546604 | 0.495654 | 0.51425 | 0.5007 |
| **144** | 1.9475 | 2.0275 | 0.535062 | 0.480937 | 0.53078 | 0.50047 |
| **145** | 1.875 | 2.0546 | 0.534884 | 0.515891 | 0.53455 | 0.50836 |
| **146** | 1.8722 | 1.9963 | 0.529228 | 0.517342 | 0.51102 | 0.5059 |
| **147** | 1.9038 | 1.968 | 0.518983 | 0.493185 | 0.50695 | 0.51878 |
| **148** | 1.837 | 1.9549 | 0.530454 | 0.516515 | 0.50579 | 0.53875 |
| **149** | 1.8487 | 1.9733 | 0.520751 | 0.490284 | 0.52042 | 0.52321 |
| **150** | 1.9028 | 2.0036 | 0.544298 | 0.538166 | 0.52783 | 0.52026 |
| **151** | 1.8613 | 1.9593 | 0.565218 | 0.517453 | 0.53326 | 0.53037 |
| **152** | 1.8304 | 2.0249 | 0.538646 | 0.507834 | 0.50891 | 0.54458 |
| **153** | 1.8501 | 1.9548 | 0.525102 | 0.555874 | 0.50191 | 0.5471 |
| **154** | 1.8417 | 2.0359 | 0.529565 | 0.527205 | 0.51477 | 0.52465 |
| **155** | 1.9434 | 2.0446 | 0.513164 | 0.543569 | 0.49198 | 0.54513 |
| **156** | 1.8932 | 1.9993 | 0.541555 | 0.583721 | 0.53299 | 0.55726 |
| **157** | 1.8273 | 1.9795 | 0.547131 | 0.53777 | 0.54102 | 0.54242 |
| **158** | 1.884 | 1.9531 | 0.512131 | 0.519044 | 0.50023 | 0.50736 |
| **159** | 1.8738 | 1.9669 | 0.541874 | 0.562109 | 0.52736 | 0.53384 |
| **160** | 1.8725 | 1.9021 | 0.547687 | 0.53 | 0.52594 | 0.54801 |
| **161** | 1.8518 | 1.9262 | 0.555831 | 0.519127 | 0.54433 | 0.56715 |
| **162** | 1.8281 | 1.9157 | 0.548648 | 0.515436 | 0.53188 | 0.5409 |
| **163** | 1.8896 | 1.9505 | 0.534253 | 0.518325 | 0.52671 | 0.54268 |
| **164** | 1.8298 | 1.9207 | 0.559967 | 0.531013 | 0.51904 | 0.52938 |
| **165** | 1.8243 | 1.8928 | 0.566332 | 0.543304 | 0.54194 | 0.51622 |
| **166** | 1.8482 | 1.9337 | 0.558504 | 0.512895 | 0.53942 | 0.5152 |
| **167** | 1.8289 | 1.9492 | 0.543606 | 0.554773 | 0.54763 | 0.55411 |
| **168** | 1.8376 | 1.935 | 0.546074 | 0.572086 | 0.53311 | 0.56046 |
| **169** | 1.7952 | 1.8945 | 0.550914 | 0.563152 | 0.55372 | 0.56268 |
| **170** | 1.8828 | 1.9042 | 0.546035 | 0.514232 | 0.5495 | 0.55367 |
| **171** | 1.8812 | 1.9063 | 0.554366 | 0.555157 | 0.53988 | 0.56439 |
| **172** | 1.9043 | 1.9657 | 0.561836 | 0.539528 | 0.52518 | 0.54829 |
| **173** | 1.8554 | 1.9735 | 0.531494 | 0.553465 | 0.54807 | 0.5633 |
| **174** | 1.8428 | 1.977 | 0.534765 | 0.548908 | 0.53065 | 0.55619 |
| **175** | 1.8176 | 1.9488 | 0.550518 | 0.540161 | 0.54441 | 0.55699 |
| **176** | 1.8405 | 1.9842 | 0.536144 | 0.56265 | 0.52691 | 0.55976 |
| **177** | 1.8435 | 1.9529 | 0.530742 | 0.522223 | 0.52705 | 0.54436 |
| **178** | 1.8688 | 1.9772 | 0.567862 | 0.536401 | 0.55032 | 0.53643 |
| **179** | 1.8528 | 1.9851 | 0.556689 | 0.575906 | 0.55428 | 0.5581 |
| **180** | 1.8134 | 1.9735 | 0.564311 | 0.546638 | 0.55159 | 0.54155 |
| **181** | 1.8361 | 1.9501 | 0.56436 | 0.571679 | 0.54836 | 0.55458 |
| **182** | 1.8119 | 1.9675 | 0.56135 | 0.547561 | 0.55085 | 0.54193 |
| **183** | 1.7878 | 1.9206 | 0.563831 | 0.541652 | 0.55197 | 0.53048 |
| **184** | 1.8192 | 1.9279 | 0.566044 | 0.537762 | 0.55451 | 0.55124 |
| **185** | 1.8052 | 1.9162 | 0.568939 | 0.556477 | 0.54654 | 0.56346 |
| **186** | 1.7652 | 1.8897 | 0.553548 | 0.55531 | 0.53688 | 0.5686 |
| **187** | 1.8051 | 1.9385 | 0.545948 | 0.540967 | 0.53711 | 0.56018 |
| **188** | 1.8425 | 1.9323 | 0.55248 | 0.529024 | 0.53484 | 0.54594 |
| **189** | 1.7989 | 1.9062 | 0.55491 | 0.556892 | 0.53844 | 0.55796 |
| **190** | 1.8025 | 1.8856 | 0.573559 | 0.557163 | 0.53855 | 0.57223 |
| **191** | 1.8827 | 1.9459 | 0.552399 | 0.532375 | 0.52532 | 0.55561 |
| **192** | 1.8361 | 1.9173 | 0.556354 | 0.561764 | 0.52326 | 0.55426 |
| **193** | 1.834 | 1.8837 | 0.57039 | 0.539316 | 0.53497 | 0.54632 |
| **194** | 1.8203 | 1.8719 | 0.553099 | 0.553417 | 0.53587 | 0.56526 |
| **195** | 1.8411 | 1.8856 | 0.565574 | 0.53349 | 0.53788 | 0.55437 |
| **196** | 1.8198 | 1.8805 | 0.569457 | 0.534 | 0.53436 | 0.5482 |
| **197** | 1.8127 | 1.8778 | 0.555658 | 0.533133 | 0.53297 | 0.54834 |
| **198** | 1.8176 | 1.8895 | 0.566578 | 0.551339 | 0.53052 | 0.55138 |
| **199** | 1.82 | 1.9039 | 0.555944 | 0.552615 | 0.53309 | 0.55552 |
| **200** | 1.811 | 1.9147 | 0.572723 | 0.543085 | 0.53522 | 0.55635 |
| **201** | 1.8927 | 1.9696 | 0.564432 | 0.53747 | 0.54175 | 0.53884 |
| **202** | 1.8556 | 1.9549 | 0.566283 | 0.568933 | 0.53963 | 0.54455 |
| **203** | 1.9236 | 2.0359 | 0.518669 | 0.499145 | 0.49101 | 0.51736 |
| **204** | 1.9724 | 2.0979 | 0.508023 | 0.464652 | 0.52788 | 0.44301 |
| **205** | 2.0329 | 1.9862 | 0.501364 | 0.506824 | 0.48312 | 0.49034 |
| **206** | 1.9619 | 2.1691 | 0.526809 | 0.459875 | 0.50549 | 0.47319 |
| **207** | 2.1005 | 2.1174 | 0.529448 | 0.539125 | 0.49807 | 0.51673 |
| **208** | 2.1329 | 2.0599 | 0.468429 | 0.547861 | 0.43058 | 0.51202 |
| **209** | 1.9591 | 1.9921 | 0.536494 | 0.489466 | 0.52557 | 0.51078 |
| **210** | 1.9471 | 2.0321 | 0.537368 | 0.527943 | 0.52528 | 0.50521 |
| **211** | 2.0782 | 2.0337 | 0.49168 | 0.485982 | 0.44956 | 0.48625 |
| **212** | 2.0458 | 2.0949 | 0.446972 | 0.469912 | 0.44694 | 0.43388 |
| **213** | 2.0603 | 2.1977 | 0.501995 | 0.457703 | 0.4862 | 0.45713 |
| **214** | 1.9479 | 1.9077 | 0.531126 | 0.490314 | 0.51005 | 0.47238 |
| **215** | 1.8954 | 2.0317 | 0.554156 | 0.459514 | 0.54874 | 0.48881 |
| **216** | 2.0067 | 2.1297 | 0.536235 | 0.494193 | 0.50253 | 0.48117 |
| **217** | 2.0276 | 2.0825 | 0.5234 | 0.421163 | 0.46119 | 0.45746 |
| **218** | 2.0589 | 2.072 | 0.470359 | 0.487983 | 0.4803 | 0.45983 |
| **219** | 1.938 | 2.0406 | 0.495581 | 0.507877 | 0.46639 | 0.49236 |
| **220** | 1.8769 | 1.891 | 0.494332 | 0.505749 | 0.49104 | 0.48063 |
| **221** | 1.9566 | 1.9571 | 0.519809 | 0.475021 | 0.50736 | 0.45548 |
| **222** | 2.0372 | 1.9814 | 0.526128 | 0.469819 | 0.52049 | 0.47233 |
| **223** | 1.9268 | 2.0552 | 0.522365 | 0.460703 | 0.4955 | 0.45791 |
| **224** | 1.9586 | 1.9606 | 0.527463 | 0.52436 | 0.51584 | 0.49569 |
| **225** | 1.9483 | 1.9904 | 0.520172 | 0.482028 | 0.49961 | 0.47876 |
| **226** | 2.0005 | 1.9739 | 0.524125 | 0.505226 | 0.54491 | 0.45739 |
| **227** | 1.9416 | 2.0281 | 0.537006 | 0.542918 | 0.52457 | 0.50996 |
| **228** | 1.8718 | 2.0219 | 0.523718 | 0.498408 | 0.51773 | 0.43865 |
| **229** | 1.9282 | 2.0271 | 0.522916 | 0.501367 | 0.53397 | 0.4512 |
| **230** | 1.9147 | 1.9804 | 0.518255 | 0.520046 | 0.52348 | 0.47439 |
| **231** | 2.0519 | 2.003 | 0.467654 | 0.50825 | 0.44224 | 0.49638 |
| **232** | 2.1027 | 2.0364 | 0.551733 | 0.507178 | 0.53029 | 0.47753 |
| **233** | 1.9732 | 2.0231 | 0.508615 | 0.517282 | 0.49891 | 0.46863 |
| **234** | 1.9405 | 1.9998 | 0.540527 | 0.557842 | 0.53911 | 0.5608 |
| **235** | 2.0144 | 1.9864 | 0.524659 | 0.539698 | 0.5231 | 0.50851 |
| **236** | 2.1061 | 2.025 | 0.51894 | 0.528514 | 0.54185 | 0.50065 |
| **237** | 2.0236 | 1.972 | 0.542314 | 0.493029 | 0.51968 | 0.49551 |
| **238** | 1.9447 | 2.0156 | 0.532751 | 0.501194 | 0.51793 | 0.48958 |
| **239** | 1.9376 | 1.9876 | 0.554897 | 0.481015 | 0.52028 | 0.45838 |
| **240** | 1.8397 | 2.0202 | 0.558552 | 0.51267 | 0.54732 | 0.48764 |
| **241** | 1.9484 | 1.9538 | 0.5618 | 0.524 | 0.5569 | 0.51751 |
| **242** | 1.8341 | 1.9142 | 0.561077 | 0.515419 | 0.52914 | 0.47529 |
| **243** | 2.039 | 1.916 | 0.541915 | 0.531466 | 0.5254 | 0.50297 |
| **244** | 1.911 | 1.9927 | 0.526147 | 0.521837 | 0.49417 | 0.52842 |
| **245** | 1.9534 | 2.1062 | 0.54305 | 0.539504 | 0.52337 | 0.5131 |
| **246** | 2.0001 | 1.9311 | 0.514664 | 0.555263 | 0.47873 | 0.5188 |
| **247** | 2.0182 | 1.9627 | 0.519789 | 0.515921 | 0.50891 | 0.52823 |
| **248** | 1.9101 | 1.9248 | 0.560557 | 0.558563 | 0.57048 | 0.52078 |
| **249** | 2.0406 | 2.0748 | 0.526361 | 0.542516 | 0.50895 | 0.50934 |
| **250** | 1.828 | 1.9266 | 0.553022 | 0.525829 | 0.58693 | 0.53211 |
| **251** | 1.9333 | 1.9269 | 0.527794 | 0.530686 | 0.52832 | 0.53641 |
| **252** | 1.8956 | 1.899 | 0.528069 | 0.51107 | 0.50069 | 0.50757 |
| **253** | 1.9039 | 2.0162 | 0.554915 | 0.497195 | 0.54601 | 0.47406 |
| **254** | 2.0222 | 2.0057 | 0.538393 | 0.501152 | 0.54969 | 0.51577 |
| **255** | 2.0079 | 1.9155 | 0.537719 | 0.539127 | 0.51863 | 0.53789 |
| **256** | 1.9534 | 1.9683 | 0.564926 | 0.498667 | 0.55095 | 0.52941 |
| **257** | 2.0406 | 1.9447 | 0.536607 | 0.545154 | 0.53805 | 0.55298 |
| **258** | 2.1312 | 2.0297 | 0.58101 | 0.554388 | 0.56525 | 0.5679 |
| **259** | 1.8739 | 1.9067 | 0.584076 | 0.535752 | 0.54123 | 0.56451 |
| **260** | 1.8384 | 1.9013 | 0.549496 | 0.529338 | 0.53742 | 0.51395 |
| **261** | 1.9254 | 1.9056 | 0.544254 | 0.555354 | 0.54896 | 0.54476 |
| **262** | 2.0161 | 1.9721 | 0.520109 | 0.531488 | 0.54054 | 0.51177 |
| **263** | 1.9329 | 1.8907 | 0.546786 | 0.556067 | 0.53452 | 0.55304 |
| **264** | 1.9303 | 1.9059 | 0.55113 | 0.537682 | 0.52711 | 0.52389 |
| **265** | 1.8953 | 1.9428 | 0.515451 | 0.57325 | 0.50994 | 0.56048 |
| **266** | 1.9335 | 1.9587 | 0.581751 | 0.52594 | 0.59609 | 0.54681 |
| **267** | 1.9482 | 1.9308 | 0.576457 | 0.524498 | 0.56177 | 0.54316 |
| **268** | 1.8672 | 1.9529 | 0.597361 | 0.525317 | 0.5918 | 0.53038 |
| **269** | 1.9794 | 1.9879 | 0.527646 | 0.550403 | 0.54472 | 0.53555 |
| **270** | 1.9512 | 2.0724 | 0.553416 | 0.521208 | 0.52553 | 0.49983 |
| **271** | 1.9425 | 1.9689 | 0.552543 | 0.556419 | 0.53617 | 0.5391 |
| **272** | 1.9655 | 2.0348 | 0.556156 | 0.578101 | 0.55766 | 0.58505 |
| **273** | 1.9261 | 2.029 | 0.564813 | 0.541094 | 0.57163 | 0.54318 |
| **274** | 2.0165 | 2.0519 | 0.541855 | 0.547152 | 0.51621 | 0.54252 |
| **275** | 1.8685 | 1.9966 | 0.556073 | 0.531319 | 0.55927 | 0.52705 |
| **276** | 1.9142 | 1.9155 | 0.5607 | 0.531011 | 0.53188 | 0.51765 |
| **277** | 1.9839 | 1.9889 | 0.565485 | 0.51965 | 0.52411 | 0.51264 |
| **278** | 1.8918 | 1.9313 | 0.559041 | 0.540498 | 0.57098 | 0.54389 |
| **279** | 1.9174 | 1.8843 | 0.574788 | 0.544431 | 0.56986 | 0.53777 |
| **280** | 1.897 | 1.9827 | 0.526586 | 0.567987 | 0.54868 | 0.57066 |
| **281** | 1.9282 | 1.9316 | 0.552908 | 0.58251 | 0.52643 | 0.56401 |
| **282** | 1.9405 | 2.0214 | 0.57356 | 0.549604 | 0.55677 | 0.51916 |
| **283** | 1.9437 | 1.9629 | 0.581011 | 0.545992 | 0.57141 | 0.54328 |
| **284** | 1.8335 | 1.9735 | 0.589133 | 0.586292 | 0.58088 | 0.59302 |
| **285** | 2.0185 | 1.9561 | 0.548953 | 0.543216 | 0.57447 | 0.56551 |
| **286** | 1.9665 | 1.9829 | 0.558811 | 0.557248 | 0.52232 | 0.51839 |
| **287** | 1.9211 | 2.0108 | 0.530734 | 0.57549 | 0.54893 | 0.57644 |
| **288** | 1.9416 | 1.9605 | 0.54792 | 0.586774 | 0.56355 | 0.58 |
| **289** | 1.8716 | 2.0153 | 0.57444 | 0.546274 | 0.5916 | 0.56069 |
| **290** | 1.8938 | 1.9658 | 0.592676 | 0.557891 | 0.61496 | 0.54784 |
| **291** | 1.9925 | 2.0256 | 0.58276 | 0.606558 | 0.59703 | 0.57347 |
| **292** | 1.9269 | 1.9374 | 0.559649 | 0.561272 | 0.57524 | 0.55526 |
| **293** | 1.8794 | 1.9266 | 0.582113 | 0.520792 | 0.59289 | 0.537 |
| **294** | 1.9056 | 1.9198 | 0.550957 | 0.505315 | 0.57239 | 0.49504 |
| **295** | 1.8854 | 1.9915 | 0.555328 | 0.534974 | 0.54377 | 0.52082 |
| **296** | 1.8884 | 1.9748 | 0.53678 | 0.57319 | 0.54616 | 0.57771 |
| **297** | 1.853 | 1.958 | 0.582962 | 0.55936 | 0.58652 | 0.5628 |
| **298** | 1.9172 | 1.9565 | 0.587192 | 0.566353 | 0.5864 | 0.55487 |
| **299** | 1.9219 | 1.9725 | 0.578565 | 0.570515 | 0.60601 | 0.5712 |
| **300** | 1.9001 | 1.911 | 0.520471 | 0.549349 | 0.54006 | 0.54398 |
| **301** | 1.8806 | 1.8963 | 0.532442 | 0.560499 | 0.57666 | 0.54625 |
| **302** | 1.8188 | 1.9032 | 0.558781 | 0.566286 | 0.58578 | 0.54031 |
| **303** | 1.8097 | 1.9062 | 0.581379 | 0.548455 | 0.56637 | 0.53808 |
| **304** | 1.7988 | 1.9402 | 0.564315 | 0.568951 | 0.55314 | 0.56335 |
| **305** | 1.8703 | 1.9601 | 0.579368 | 0.589588 | 0.5918 | 0.60392 |
| **306** | 1.8471 | 1.934 | 0.551388 | 0.568128 | 0.61123 | 0.5994 |
| **307** | 1.7837 | 1.9299 | 0.538685 | 0.586764 | 0.55109 | 0.57904 |
| **308** | 1.8321 | 1.9171 | 0.537729 | 0.554635 | 0.55488 | 0.55767 |
| **309** | 1.7873 | 1.9201 | 0.553293 | 0.561949 | 0.58619 | 0.56037 |
| **310** | 1.7897 | 1.8787 | 0.605883 | 0.569688 | 0.63373 | 0.56986 |
| **311** | 1.8285 | 1.8985 | 0.638316 | 0.588215 | 0.63836 | 0.56898 |
| **312** | 1.8956 | 1.9464 | 0.635935 | 0.550837 | 0.63771 | 0.56826 |
| **313** | 1.849 | 1.9613 | 0.621817 | 0.557502 | 0.63781 | 0.53268 |
| **314** | 1.8136 | 1.9032 | 0.61115 | 0.577322 | 0.62498 | 0.52939 |
| **315** | 1.8385 | 1.9686 | 0.597641 | 0.538043 | 0.61581 | 0.51531 |
| **316** | 1.8093 | 1.916 | 0.592296 | 0.564965 | 0.62177 | 0.54185 |
| **317** | 1.8399 | 1.9629 | 0.614625 | 0.558613 | 0.6297 | 0.53691 |
| **318** | 1.8225 | 1.9579 | 0.563648 | 0.583857 | 0.56179 | 0.55741 |
| **319** | 1.7541 | 1.8675 | 0.600383 | 0.581618 | 0.60497 | 0.60723 |
| **320** | 1.7848 | 1.9425 | 0.570124 | 0.58704 | 0.59018 | 0.59433 |
| **321** | 1.8512 | 1.9792 | 0.574552 | 0.60759 | 0.58607 | 0.60172 |
| **322** | 1.7974 | 1.9455 | 0.567906 | 0.555129 | 0.5989 | 0.58215 |
| **323** | 1.8166 | 1.8914 | 0.60172 | 0.585822 | 0.63161 | 0.57939 |
| **324** | 1.834 | 1.9293 | 0.616449 | 0.589335 | 0.63064 | 0.58866 |
| **325** | 1.8124 | 1.9013 | 0.561057 | 0.568953 | 0.6049 | 0.5824 |
| **326** | 1.8151 | 1.9243 | 0.579012 | 0.576 | 0.61373 | 0.57221 |
| **327** | 1.8177 | 1.9417 | 0.570229 | 0.542605 | 0.58104 | 0.556 |
| **328** | 1.8073 | 1.9346 | 0.582083 | 0.576544 | 0.5751 | 0.58605 |
| **329** | 1.8455 | 1.8827 | 0.580287 | 0.560972 | 0.59549 | 0.572 |
| **330** | 1.8179 | 1.901 | 0.57976 | 0.588739 | 0.60084 | 0.56583 |
| **331** | 1.8059 | 1.8752 | 0.600328 | 0.586538 | 0.58424 | 0.5866 |
| **332** | 1.7606 | 1.9361 | 0.598863 | 0.544601 | 0.60502 | 0.5378 |
| **333** | 1.7705 | 1.8956 | 0.598352 | 0.558093 | 0.57714 | 0.54598 |
| **334** | 1.7765 | 1.8837 | 0.58351 | 0.550244 | 0.59996 | 0.54825 |
| **335** | 1.816 | 1.9049 | 0.592635 | 0.533324 | 0.6057 | 0.54478 |
| **336** | 1.8236 | 1.987 | 0.609778 | 0.584287 | 0.62454 | 0.59152 |
| **337** | 1.7711 | 1.8954 | 0.620064 | 0.578522 | 0.62031 | 0.59453 |
| **338** | 1.8428 | 1.906 | 0.609652 | 0.610895 | 0.61894 | 0.60135 |
| **339** | 1.8378 | 1.9126 | 0.628638 | 0.594324 | 0.61782 | 0.59897 |
| **340** | 1.8048 | 1.9063 | 0.614689 | 0.558159 | 0.62959 | 0.56146 |
| **341** | 1.761 | 1.8915 | 0.6177 | 0.570353 | 0.62517 | 0.56043 |
| **342** | 1.8175 | 1.8839 | 0.59971 | 0.570257 | 0.60649 | 0.57625 |
| **343** | 1.8142 | 1.9056 | 0.590623 | 0.595511 | 0.60519 | 0.5859 |
| **344** | 1.7901 | 1.918 | 0.586103 | 0.567632 | 0.59618 | 0.56658 |
| **345** | 1.8003 | 1.8996 | 0.588103 | 0.558627 | 0.60838 | 0.55332 |
| **346** | 1.8116 | 1.8665 | 0.612557 | 0.564048 | 0.61178 | 0.55821 |
| **347** | 1.8085 | 1.8614 | 0.606063 | 0.560945 | 0.60442 | 0.5799 |
| **348** | 1.7744 | 1.9135 | 0.613246 | 0.562288 | 0.61866 | 0.56003 |
| **349** | 1.7958 | 1.8997 | 0.610309 | 0.575384 | 0.63518 | 0.5839 |
| **350** | 1.8389 | 1.8756 | 0.597298 | 0.574646 | 0.61431 | 0.57105 |
| **351** | 1.7703 | 1.8884 | 0.594304 | 0.570129 | 0.62873 | 0.58218 |
| **352** | 1.7679 | 1.9218 | 0.595803 | 0.581647 | 0.63632 | 0.58123 |
| **353** | 1.7669 | 1.9018 | 0.600192 | 0.59001 | 0.61848 | 0.57166 |
| **354** | 1.8034 | 1.843 | 0.586108 | 0.569111 | 0.62316 | 0.57074 |
| **355** | 1.8248 | 1.8691 | 0.59073 | 0.594468 | 0.6087 | 0.59678 |
| **356** | 1.7698 | 1.8611 | 0.6037 | 0.616439 | 0.62045 | 0.60226 |
| **357** | 1.7494 | 1.8673 | 0.610699 | 0.569311 | 0.62745 | 0.58208 |
| **358** | 1.7887 | 1.8676 | 0.593161 | 0.55835 | 0.60533 | 0.54557 |
| **359** | 1.7617 | 1.8929 | 0.601749 | 0.55399 | 0.61599 | 0.55837 |
| **360** | 1.7806 | 1.8761 | 0.623411 | 0.578999 | 0.65689 | 0.5731 |
| **361** | 1.7543 | 1.8952 | 0.593925 | 0.565231 | 0.61122 | 0.56534 |
| **362** | 1.7874 | 1.8938 | 0.590402 | 0.567529 | 0.61474 | 0.57955 |
| **363** | 1.7756 | 1.8669 | 0.598574 | 0.565564 | 0.60469 | 0.57571 |
| **364** | 1.7687 | 1.826 | 0.578081 | 0.548549 | 0.60458 | 0.56558 |
| **365** | 1.7669 | 1.8947 | 0.597977 | 0.577301 | 0.60752 | 0.56188 |
| **366** | 1.7949 | 1.899 | 0.595027 | 0.557684 | 0.59871 | 0.55788 |
| **367** | 1.774 | 1.8597 | 0.598418 | 0.592575 | 0.61009 | 0.58654 |
| **368** | 1.7831 | 1.8359 | 0.592161 | 0.595935 | 0.61422 | 0.57504 |
| **369** | 1.7726 | 1.8681 | 0.623791 | 0.598795 | 0.6259 | 0.57925 |
| **370** | 1.7935 | 1.8594 | 0.613017 | 0.587329 | 0.62949 | 0.59477 |
| **371** | 1.7775 | 1.9609 | 0.592132 | 0.593481 | 0.61075 | 0.58832 |
| **372** | 1.7775 | 1.9183 | 0.603662 | 0.549245 | 0.60961 | 0.54535 |
| **373** | 1.7823 | 1.9255 | 0.610043 | 0.556254 | 0.64256 | 0.57047 |
| **374** | 1.7738 | 1.9335 | 0.616821 | 0.575633 | 0.62476 | 0.57439 |
| **375** | 1.7759 | 1.9243 | 0.635111 | 0.569623 | 0.63067 | 0.56019 |
| **376** | 1.8053 | 1.885 | 0.616129 | 0.572735 | 0.62783 | 0.58263 |
| **377** | 1.8186 | 1.8998 | 0.615353 | 0.578896 | 0.62285 | 0.57687 |
| **378** | 1.8218 | 1.9064 | 0.608794 | 0.587059 | 0.62863 | 0.5772 |
| **379** | 1.8095 | 1.8872 | 0.60419 | 0.581491 | 0.61552 | 0.58563 |
| **380** | 1.7703 | 1.891 | 0.608051 | 0.589298 | 0.61481 | 0.58189 |
| **381** | 1.7907 | 1.9 | 0.602181 | 0.565926 | 0.6186 | 0.57824 |
| **382** | 1.7971 | 1.8989 | 0.612409 | 0.563886 | 0.61625 | 0.58812 |
| **383** | 1.8102 | 1.884 | 0.617774 | 0.578496 | 0.62915 | 0.58086 |
| **384** | 1.786 | 1.8729 | 0.606841 | 0.586413 | 0.60971 | 0.58471 |
| **385** | 1.7955 | 1.8911 | 0.611723 | 0.601807 | 0.62106 | 0.60048 |
| **386** | 1.7795 | 1.8508 | 0.622837 | 0.582284 | 0.64274 | 0.60923 |
| **387** | 1.7783 | 1.8499 | 0.623423 | 0.583529 | 0.64317 | 0.59681 |
| **388** | 1.7763 | 1.8709 | 0.604807 | 0.567897 | 0.61034 | 0.5871 |
| **389** | 1.7666 | 1.8535 | 0.610762 | 0.563134 | 0.62288 | 0.58635 |
| **390** | 1.7617 | 1.8341 | 0.610369 | 0.567576 | 0.61113 | 0.5934 |
| **391** | 1.7559 | 1.8963 | 0.599182 | 0.594595 | 0.60573 | 0.60316 |
| **392** | 1.7328 | 1.8919 | 0.614114 | 0.579307 | 0.61573 | 0.59885 |
| **393** | 1.7376 | 1.8733 | 0.597037 | 0.592429 | 0.61902 | 0.59877 |
| **394** | 1.7326 | 1.8749 | 0.581448 | 0.596551 | 0.63325 | 0.59946 |
| **395** | 1.7523 | 1.8717 | 0.593674 | 0.602197 | 0.61986 | 0.60876 |
| **396** | 1.7552 | 1.8718 | 0.599081 | 0.593088 | 0.61975 | 0.60908 |
| **397** | 1.7632 | 1.8661 | 0.597205 | 0.594738 | 0.62164 | 0.61234 |
| **398** | 1.7456 | 1.8851 | 0.601624 | 0.591606 | 0.61638 | 0.61134 |
| **399** | 1.7431 | 1.9151 | 0.606337 | 0.58633 | 0.6182 | 0.61138 |
| **400** | 1.7391 | 1.9039 | 0.596255 | 0.588651 | 0.61986 | 0.61102 |
| **401** | 1.7996 | 1.8876 | 0.588741 | 0.583593 | 0.6266 | 0.59213 |
| **402** | 1.8196 | 1.8836 | 0.624545 | 0.591629 | 0.62028 | 0.57992 |
| **403** | 1.8782 | 1.9341 | 0.555382 | 0.548754 | 0.56546 | 0.55432 |
| **404** | 1.8519 | 2.0275 | 0.55458 | 0.541016 | 0.55472 | 0.53331 |
| **405** | 1.9637 | 1.9498 | 0.566602 | 0.576611 | 0.58239 | 0.54553 |
| **406** | 1.9928 | 2.0247 | 0.593366 | 0.553801 | 0.60111 | 0.53262 |
| **407** | 1.838 | 1.9262 | 0.547026 | 0.550642 | 0.59217 | 0.55698 |
| **408** | 1.9002 | 1.9902 | 0.559945 | 0.568148 | 0.58277 | 0.54523 |
| **409** | 1.7855 | 1.8978 | 0.655286 | 0.526387 | 0.65211 | 0.55476 |
| **410** | 1.9094 | 2.0213 | 0.599498 | 0.611844 | 0.61546 | 0.60955 |
| **411** | 1.8383 | 2.05 | 0.594129 | 0.520762 | 0.61019 | 0.49533 |
| **412** | 1.8812 | 1.9785 | 0.582805 | 0.531887 | 0.57188 | 0.47954 |
| **413** | 1.9842 | 2.0101 | 0.524814 | 0.550532 | 0.53353 | 0.56881 |
| **414** | 1.9112 | 2.0162 | 0.572195 | 0.508775 | 0.60777 | 0.50102 |
| **415** | 1.9273 | 1.9588 | 0.569481 | 0.597761 | 0.58038 | 0.58536 |
| **416** | 1.8817 | 2.031 | 0.564543 | 0.583788 | 0.56967 | 0.56917 |
| **417** | 1.8362 | 1.9657 | 0.535891 | 0.530478 | 0.57286 | 0.55514 |
| **418** | 1.813 | 2.028 | 0.561803 | 0.557127 | 0.58814 | 0.54059 |
| **419** | 1.9377 | 1.9143 | 0.565194 | 0.563078 | 0.57614 | 0.56779 |
| **420** | 1.853 | 1.9188 | 0.576854 | 0.584476 | 0.58495 | 0.57332 |
| **421** | 1.9089 | 1.9852 | 0.597036 | 0.533834 | 0.56741 | 0.53776 |
| **422** | 1.8761 | 1.9762 | 0.596588 | 0.551866 | 0.58509 | 0.55778 |
| **423** | 1.7955 | 2.0486 | 0.600851 | 0.564309 | 0.54515 | 0.54546 |
| **424** | 1.8704 | 2.011 | 0.55979 | 0.54048 | 0.55408 | 0.52774 |
| **425** | 1.781 | 1.919 | 0.588993 | 0.516715 | 0.55044 | 0.50884 |
| **426** | 1.814 | 1.922 | 0.539895 | 0.569568 | 0.52637 | 0.56415 |
| **427** | 1.8696 | 2.0379 | 0.607546 | 0.537467 | 0.60213 | 0.51662 |
| **428** | 1.9016 | 1.911 | 0.581163 | 0.554821 | 0.5845 | 0.52692 |
| **429** | 1.9458 | 1.9548 | 0.555249 | 0.507909 | 0.59141 | 0.54149 |
| **430** | 1.9305 | 2.1183 | 0.601324 | 0.545993 | 0.59443 | 0.49847 |
| **431** | 1.9129 | 2.122 | 0.574787 | 0.531439 | 0.57519 | 0.50178 |
| **432** | 1.8667 | 1.9445 | 0.568439 | 0.52971 | 0.5853 | 0.51717 |
| **433** | 1.8612 | 1.9283 | 0.551152 | 0.512837 | 0.54465 | 0.53132 |
| **434** | 1.8131 | 2.0173 | 0.535865 | 0.516808 | 0.55856 | 0.5339 |
| **435** | 1.9154 | 1.9699 | 0.600908 | 0.562314 | 0.63285 | 0.55886 |
| **436** | 1.8782 | 1.9623 | 0.589049 | 0.554257 | 0.59129 | 0.55021 |
| **437** | 1.8995 | 1.9263 | 0.567253 | 0.566821 | 0.57551 | 0.5648 |
| **438** | 1.9433 | 2.0932 | 0.537548 | 0.527435 | 0.54607 | 0.51668 |
| **439** | 1.8955 | 2.0216 | 0.556882 | 0.550504 | 0.54073 | 0.5082 |
| **440** | 1.9462 | 1.8834 | 0.562214 | 0.551459 | 0.54036 | 0.53926 |
| **441** | 1.8588 | 1.9518 | 0.592856 | 0.548711 | 0.57391 | 0.53277 |
| **442** | 1.9301 | 1.8865 | 0.553616 | 0.544221 | 0.57017 | 0.52261 |
| **443** | 1.9422 | 1.9422 | 0.542867 | 0.571257 | 0.53555 | 0.56607 |
| **444** | 1.8703 | 1.9172 | 0.561696 | 0.548858 | 0.55225 | 0.55736 |
| **445** | 2.0413 | 2.0903 | 0.584809 | 0.571537 | 0.56789 | 0.54223 |
| **446** | 1.9326 | 1.9874 | 0.554719 | 0.539121 | 0.537 | 0.51156 |
| **447** | 1.8697 | 2.0274 | 0.563915 | 0.554194 | 0.57194 | 0.53262 |
| **448** | 2.0148 | 1.9845 | 0.62283 | 0.568351 | 0.61734 | 0.55289 |
| **449** | 2.0069 | 2.0344 | 0.586416 | 0.540401 | 0.60585 | 0.5166 |
| **450** | 1.8536 | 1.951 | 0.574487 | 0.569287 | 0.60855 | 0.55139 |
| **451** | 1.8721 | 1.938 | 0.597774 | 0.541937 | 0.5927 | 0.54092 |
| **452** | 1.8903 | 1.9493 | 0.590247 | 0.543236 | 0.58876 | 0.53305 |
| **453** | 1.837 | 1.9924 | 0.59955 | 0.560579 | 0.62477 | 0.53566 |
| **454** | 1.8108 | 2.0199 | 0.561538 | 0.51973 | 0.55766 | 0.51241 |
| **455** | 1.8334 | 1.8934 | 0.563998 | 0.561057 | 0.54469 | 0.55725 |
| **456** | 1.8284 | 1.9792 | 0.590271 | 0.568377 | 0.58385 | 0.57422 |
| **457** | 1.9488 | 1.9429 | 0.604406 | 0.550403 | 0.62346 | 0.54748 |
| **458** | 1.8777 | 1.989 | 0.629124 | 0.550623 | 0.64952 | 0.52221 |
| **459** | 1.796 | 1.9273 | 0.590695 | 0.545738 | 0.62092 | 0.54462 |
| **460** | 1.7838 | 1.9219 | 0.555625 | 0.564052 | 0.55248 | 0.54091 |
| **461** | 1.8545 | 1.9451 | 0.546112 | 0.594646 | 0.56188 | 0.56047 |
| **462** | 1.9078 | 1.9835 | 0.538344 | 0.564036 | 0.54931 | 0.5338 |
| **463** | 1.895 | 1.9606 | 0.614179 | 0.565716 | 0.60071 | 0.58175 |
| **464** | 1.8184 | 1.9415 | 0.584713 | 0.553909 | 0.56856 | 0.5102 |
| **465** | 1.8439 | 1.8808 | 0.591465 | 0.555266 | 0.59195 | 0.5368 |
| **466** | 1.9435 | 1.9191 | 0.567861 | 0.605973 | 0.59899 | 0.60074 |
| **467** | 1.9327 | 2.0162 | 0.572846 | 0.597396 | 0.57817 | 0.58225 |
| **468** | 1.8815 | 1.9048 | 0.577219 | 0.57805 | 0.58953 | 0.58364 |
| **469** | 1.8601 | 1.9997 | 0.582646 | 0.621956 | 0.5855 | 0.61496 |
| **470** | 1.8705 | 1.9931 | 0.592277 | 0.568634 | 0.58268 | 0.54671 |
| **471** | 1.8535 | 1.9079 | 0.587226 | 0.562598 | 0.57095 | 0.55082 |
| **472** | 1.9811 | 2.0359 | 0.646116 | 0.609542 | 0.62525 | 0.62568 |
| **473** | 1.8897 | 1.9213 | 0.600772 | 0.609098 | 0.61339 | 0.62272 |
| **474** | 1.9279 | 1.9564 | 0.55933 | 0.586705 | 0.57037 | 0.59348 |
| **475** | 1.9359 | 1.9258 | 0.567934 | 0.572382 | 0.53053 | 0.59482 |
| **476** | 1.9248 | 1.9303 | 0.586939 | 0.572396 | 0.54358 | 0.56267 |
| **477** | 1.8968 | 1.9993 | 0.581551 | 0.539555 | 0.57118 | 0.52981 |
| **478** | 1.9173 | 1.9975 | 0.609856 | 0.592091 | 0.64065 | 0.60531 |
| **479** | 1.854 | 2.0141 | 0.605827 | 0.601306 | 0.6126 | 0.59126 |
| **480** | 1.8203 | 1.9842 | 0.615803 | 0.624481 | 0.607 | 0.6157 |
| **481** | 1.8536 | 1.94 | 0.573303 | 0.575408 | 0.59761 | 0.58623 |
| **482** | 1.8467 | 1.952 | 0.614881 | 0.573015 | 0.60719 | 0.58422 |
| **483** | 1.7926 | 1.9362 | 0.599926 | 0.606573 | 0.6053 | 0.58643 |
| **484** | 1.8222 | 1.9467 | 0.551601 | 0.582541 | 0.57734 | 0.58662 |
| **485** | 1.9545 | 1.9933 | 0.559993 | 0.599165 | 0.58574 | 0.59529 |
| **486** | 1.8836 | 1.992 | 0.569668 | 0.584847 | 0.56183 | 0.59074 |
| **487** | 1.9514 | 2.0133 | 0.550255 | 0.521586 | 0.59081 | 0.58412 |
| **488** | 1.8971 | 1.9899 | 0.587228 | 0.578225 | 0.61087 | 0.60965 |
| **489** | 1.7927 | 1.978 | 0.588955 | 0.540968 | 0.61011 | 0.56865 |
| **490** | 1.8997 | 1.9651 | 0.604025 | 0.580237 | 0.6156 | 0.587 |
| **491** | 1.835 | 2.047 | 0.5774 | 0.594888 | 0.59186 | 0.5911 |
| **492** | 1.9676 | 1.9807 | 0.577616 | 0.610912 | 0.60045 | 0.58368 |
| **493** | 1.8819 | 1.9518 | 0.578217 | 0.567637 | 0.59029 | 0.58538 |
| **494** | 1.8842 | 1.9274 | 0.57551 | 0.58622 | 0.55977 | 0.58867 |
| **495** | 1.8499 | 1.8919 | 0.601609 | 0.581031 | 0.62631 | 0.57615 |
| **496** | 1.8182 | 1.9124 | 0.591193 | 0.575013 | 0.58647 | 0.57298 |
| **497** | 1.7969 | 1.9144 | 0.591888 | 0.578626 | 0.62031 | 0.59638 |
| **498** | 1.8284 | 1.9516 | 0.628962 | 0.581028 | 0.6342 | 0.58773 |
| **499** | 1.8427 | 1.8544 | 0.601974 | 0.567291 | 0.64321 | 0.61275 |
| **500** | 1.8824 | 1.8378 | 0.58192 | 0.593433 | 0.61072 | 0.59683 |
| **501** | 1.7808 | 1.8571 | 0.558947 | 0.578986 | 0.60045 | 0.55819 |
| **502** | 1.7695 | 1.8939 | 0.601855 | 0.567483 | 0.61327 | 0.55927 |
| **503** | 1.859 | 1.9086 | 0.595954 | 0.566064 | 0.58011 | 0.56826 |
| **504** | 1.8569 | 1.8887 | 0.585364 | 0.580576 | 0.59449 | 0.59455 |
| **505** | 1.8407 | 1.9353 | 0.59437 | 0.593162 | 0.58254 | 0.5935 |
| **506** | 1.7873 | 1.8804 | 0.600734 | 0.570425 | 0.5938 | 0.57685 |
| **507** | 1.8219 | 1.8602 | 0.573852 | 0.589314 | 0.54445 | 0.57265 |
| **508** | 1.8452 | 1.9246 | 0.587119 | 0.55842 | 0.59573 | 0.54166 |
| **509** | 1.7616 | 1.9187 | 0.62108 | 0.556729 | 0.6521 | 0.52609 |
| **510** | 1.7951 | 1.9339 | 0.619057 | 0.615675 | 0.61871 | 0.59226 |
| **511** | 1.8502 | 1.9313 | 0.643694 | 0.615313 | 0.6447 | 0.57616 |
| **512** | 1.8785 | 1.9318 | 0.664765 | 0.589566 | 0.66223 | 0.58793 |
| **513** | 1.8103 | 1.9296 | 0.611876 | 0.588274 | 0.64961 | 0.56565 |
| **514** | 1.7825 | 1.8693 | 0.641222 | 0.573862 | 0.65595 | 0.56304 |
| **515** | 1.8143 | 1.9158 | 0.606461 | 0.567008 | 0.63245 | 0.54648 |
| **516** | 1.7752 | 1.8418 | 0.602478 | 0.577559 | 0.61167 | 0.55702 |
| **517** | 1.7768 | 1.946 | 0.633995 | 0.579238 | 0.63018 | 0.55162 |
| **518** | 1.7956 | 2.0312 | 0.616461 | 0.583839 | 0.61521 | 0.55921 |
| **519** | 1.7919 | 1.8914 | 0.615726 | 0.589952 | 0.62238 | 0.5894 |
| **520** | 1.8329 | 1.9524 | 0.662058 | 0.571755 | 0.63341 | 0.5467 |
| **521** | 1.7934 | 1.9385 | 0.639441 | 0.585748 | 0.65169 | 0.60207 |
| **522** | 1.7784 | 1.9513 | 0.652797 | 0.576377 | 0.63935 | 0.58524 |
| **523** | 1.7919 | 1.9273 | 0.634462 | 0.593819 | 0.63965 | 0.5517 |
| **524** | 1.7829 | 1.8866 | 0.632554 | 0.578527 | 0.63348 | 0.56208 |
| **525** | 1.766 | 1.9117 | 0.611466 | 0.592472 | 0.63897 | 0.57423 |
| **526** | 1.7258 | 1.9031 | 0.590714 | 0.588689 | 0.62319 | 0.58417 |
| **527** | 1.765 | 1.8609 | 0.615067 | 0.570757 | 0.63575 | 0.57334 |
| **528** | 1.781 | 1.9078 | 0.606015 | 0.585545 | 0.62856 | 0.59836 |
| **529** | 1.7854 | 1.8933 | 0.587782 | 0.584234 | 0.61962 | 0.59909 |
| **530** | 1.782 | 1.8165 | 0.598467 | 0.607992 | 0.60106 | 0.58977 |
| **531** | 1.7794 | 1.8571 | 0.605184 | 0.580674 | 0.6231 | 0.58716 |
| **532** | 1.8091 | 1.9302 | 0.602383 | 0.56742 | 0.5964 | 0.57147 |
| **533** | 1.7567 | 1.9408 | 0.607084 | 0.567454 | 0.63136 | 0.54581 |
| **534** | 1.7466 | 1.8914 | 0.640314 | 0.577416 | 0.6474 | 0.56349 |
| **535** | 1.76 | 1.9274 | 0.615421 | 0.570984 | 0.64087 | 0.5528 |
| **536** | 1.8373 | 1.9289 | 0.602962 | 0.579448 | 0.63604 | 0.56392 |
| **537** | 1.7798 | 1.9163 | 0.609264 | 0.583725 | 0.62191 | 0.59682 |
| **538** | 1.8209 | 1.8753 | 0.606542 | 0.597939 | 0.62467 | 0.57671 |
| **539** | 1.8204 | 1.8903 | 0.632235 | 0.58048 | 0.64727 | 0.58161 |
| **540** | 1.7947 | 1.9449 | 0.63563 | 0.588462 | 0.63963 | 0.57135 |
| **541** | 1.7404 | 1.9275 | 0.648894 | 0.569385 | 0.64477 | 0.57022 |
| **542** | 1.7942 | 1.9124 | 0.632585 | 0.614799 | 0.6335 | 0.58808 |
| **543** | 1.7769 | 1.9641 | 0.577787 | 0.583797 | 0.61848 | 0.57288 |
| **544** | 1.7597 | 1.9647 | 0.56706 | 0.578304 | 0.59861 | 0.57686 |
| **545** | 1.7789 | 1.9654 | 0.590581 | 0.586698 | 0.63762 | 0.55787 |
| **546** | 1.7924 | 1.9296 | 0.607413 | 0.538432 | 0.6404 | 0.57829 |
| **547** | 1.7722 | 1.8904 | 0.626106 | 0.582835 | 0.6351 | 0.59783 |
| **548** | 1.7811 | 1.9388 | 0.601417 | 0.572044 | 0.63167 | 0.5752 |
| **549** | 1.7896 | 1.9044 | 0.646888 | 0.606106 | 0.66275 | 0.59475 |
| **550** | 1.825 | 1.9112 | 0.626275 | 0.605082 | 0.63457 | 0.58625 |
| **551** | 1.7655 | 1.9279 | 0.626218 | 0.584527 | 0.65729 | 0.60199 |
| **552** | 1.7133 | 1.9234 | 0.610186 | 0.598795 | 0.6621 | 0.59912 |
| **553** | 1.7665 | 1.9433 | 0.644181 | 0.592357 | 0.6613 | 0.59219 |
| **554** | 1.798 | 1.9157 | 0.608988 | 0.594916 | 0.63732 | 0.59578 |
| **555** | 1.8031 | 1.8968 | 0.598442 | 0.611472 | 0.62964 | 0.61658 |
| **556** | 1.7577 | 1.8725 | 0.643953 | 0.65372 | 0.65711 | 0.63847 |
| **557** | 1.7448 | 1.931 | 0.621048 | 0.600032 | 0.64917 | 0.62027 |
| **558** | 1.7182 | 1.8868 | 0.608703 | 0.552751 | 0.62286 | 0.56817 |
| **559** | 1.7005 | 1.9979 | 0.625267 | 0.546058 | 0.65039 | 0.55183 |
| **560** | 1.7066 | 1.976 | 0.640052 | 0.590632 | 0.66829 | 0.57604 |
| **561** | 1.7119 | 1.9032 | 0.615415 | 0.59485 | 0.64385 | 0.57911 |
| **562** | 1.7438 | 1.9139 | 0.606532 | 0.585875 | 0.66128 | 0.58573 |
| **563** | 1.7329 | 1.8556 | 0.60049 | 0.57006 | 0.62652 | 0.59136 |
| **564** | 1.6927 | 1.8562 | 0.604702 | 0.580463 | 0.63466 | 0.56913 |
| **565** | 1.6872 | 1.9398 | 0.6182 | 0.580446 | 0.64517 | 0.55694 |
| **566** | 1.7337 | 1.9195 | 0.61558 | 0.571208 | 0.62711 | 0.57197 |
| **567** | 1.6986 | 1.916 | 0.612848 | 0.600995 | 0.6365 | 0.58775 |
| **568** | 1.7394 | 1.8832 | 0.616999 | 0.581288 | 0.64904 | 0.58136 |
| **569** | 1.7148 | 1.8897 | 0.646676 | 0.590269 | 0.66654 | 0.59451 |
| **570** | 1.7185 | 1.9205 | 0.651069 | 0.573847 | 0.67853 | 0.59616 |
| **571** | 1.7272 | 1.9301 | 0.623674 | 0.579757 | 0.66192 | 0.60981 |
| **572** | 1.7549 | 1.9392 | 0.62213 | 0.583339 | 0.62432 | 0.58269 |
| **573** | 1.7381 | 1.9419 | 0.61747 | 0.579977 | 0.63827 | 0.59679 |
| **574** | 1.7272 | 1.9054 | 0.641378 | 0.581925 | 0.65632 | 0.59825 |
| **575** | 1.7501 | 1.9132 | 0.627887 | 0.566615 | 0.64688 | 0.59758 |
| **576** | 1.7302 | 1.9305 | 0.675947 | 0.598929 | 0.68014 | 0.61495 |
| **577** | 1.7354 | 1.9511 | 0.647882 | 0.588758 | 0.66867 | 0.60141 |
| **578** | 1.7656 | 1.9168 | 0.639478 | 0.58658 | 0.65335 | 0.6053 |
| **579** | 1.7414 | 1.9181 | 0.647932 | 0.594267 | 0.66068 | 0.60988 |
| **580** | 1.7133 | 1.8934 | 0.635956 | 0.608489 | 0.66174 | 0.60946 |
| **581** | 1.721 | 1.9041 | 0.646143 | 0.588991 | 0.6717 | 0.57825 |
| **582** | 1.7238 | 1.9057 | 0.645985 | 0.571059 | 0.66984 | 0.5798 |
| **583** | 1.7436 | 1.8991 | 0.649768 | 0.582835 | 0.66568 | 0.59557 |
| **584** | 1.7332 | 1.9055 | 0.644362 | 0.609837 | 0.66521 | 0.61411 |
| **585** | 1.7236 | 1.8928 | 0.641554 | 0.614773 | 0.66747 | 0.61982 |
| **586** | 1.7216 | 1.9063 | 0.634723 | 0.594629 | 0.67123 | 0.61683 |
| **587** | 1.7261 | 1.9247 | 0.632599 | 0.59972 | 0.65977 | 0.61232 |
| **588** | 1.739 | 1.902 | 0.63086 | 0.591052 | 0.65517 | 0.60407 |
| **589** | 1.7467 | 1.9084 | 0.623831 | 0.59307 | 0.66402 | 0.60287 |
| **590** | 1.7321 | 1.8478 | 0.627141 | 0.57226 | 0.6648 | 0.60772 |
| **591** | 1.7568 | 1.8846 | 0.640372 | 0.57493 | 0.67314 | 0.61644 |
| **592** | 1.7526 | 1.8801 | 0.636781 | 0.598254 | 0.66069 | 0.61378 |
| **593** | 1.7555 | 1.8838 | 0.63785 | 0.586086 | 0.65301 | 0.61644 |
| **594** | 1.7488 | 1.888 | 0.62895 | 0.583808 | 0.65991 | 0.6135 |
| **595** | 1.7458 | 1.9011 | 0.638371 | 0.591007 | 0.67028 | 0.62059 |
| **596** | 1.7471 | 1.8995 | 0.637944 | 0.606925 | 0.65727 | 0.62286 |
| **597** | 1.7545 | 1.8741 | 0.640036 | 0.605386 | 0.6581 | 0.6252 |
| **598** | 1.7483 | 1.8784 | 0.63816 | 0.589196 | 0.65259 | 0.62297 |
| **599** | 1.7511 | 1.8882 | 0.637578 | 0.589123 | 0.64913 | 0.6198 |
| **600** | 1.7549 | 1.8892 | 0.635895 | 0.588241 | 0.6409 | 0.62145 |
