# Supplementary material for: WA-YOLO: An explosive material detection algorithm for blasting sites based on YOLOv8
Source: PLoS One. 2025 Apr 22;20(4):e0318172. doi: 10.1371/journal.pone.0318172 (PMC12013926; doi:10.1371/journal.pone.0318172)
Supplement: S2 Table — (DOCX) [file pone.0318172.s002.docx]

**S2 Table** Dataset Download

| **Dataset** | **Download Link** |
| --- | --- |
| **VOC 2012** | <https://host.robots.ox.ac.uk/pascal/VOC/voc2012> |
| **COCO 128** | <https://github.com/ultralytics/yolov5> |
| **Custom-built Dataset (Subset)** | <https://osf.io/h34q6/?view_only=602effb080fc466e8015fcd1b794a11a> |
